# Composite PLGA–Nanobioceramic Coating on Moxifloxacin-Loaded Akermanite 3D Porous Scaffolds for Bone Tissue Regeneration

**DOI:** 10.3390/pharmaceutics15030819

**Published:** 2023-03-02

**Authors:** Georgia K. Pouroutzidou, Lambrini Papadopoulou, Maria Lazaridou, Konstantinos Tsachouridis, Chrysanthi Papoulia, Dimitra Patsiaoura, Ioannis Tsamesidis, Konstantinos Chrissafis, George Vourlias, Konstantinos M. Paraskevopoulos, Antonios D. Anastasiou, Dimitrios N. Bikiaris, Eleana Kontonasaki

**Affiliations:** 1 Advanced Materials and Devices Laboratory, Faculty of Sciences, School of Physics, Aristotle University of Thessaloniki, 54124 Thessaloniki, Greece; 2 Department of Prosthodontics, Faculty of Health Sciences, School of Dentistry, Aristotle University of Thessaloniki, 54124 Thessaloniki, Greece; 3 School of Geology, Faculty of Sciences, Aristotle University of Thessaloniki, 54124 Thessaloniki, Greece; 4 Faculty of Sciences, School of Chemistry, Aristotle University of Thessaloniki, 54124 Thessaloniki, Greece; 5 Department of Chemical Engineering and Analytical Science, University of Manchester, Manchester M1 3AL, UK

**Keywords:** 3D porous scaffolds, foam replica technique, akermanite scaffolds, nanofillers, moxifloxacin-loaded scaffolds

## Abstract

Silica-based ceramics doped with calcium and magnesium have been proposed as suitable materials for scaffold fabrication. Akermanite (Ca_2_MgSi_2_O_7_) has attracted interest for bone regeneration due to its controllable biodegradation rate, improved mechanical properties, and high apatite-forming ability. Despite the profound advantages, ceramic scaffolds provide weak fracture resistance. The use of synthetic biopolymers such as poly(lactic-co-glycolic acid) (PLGA) as coating materials improves the mechanical performance of ceramic scaffolds and tailors their degradation rate. Moxifloxacin (MOX) is an antibiotic with antimicrobial activity against numerous aerobic and anaerobic bacteria. In this study, silica-based nanoparticles (NPs) enriched with calcium and magnesium, as well as copper and strontium ions that induce angiogenesis and osteogenesis, respectively, were incorporated into the PLGA coating. The aim was to produce composite akermanite/PLGA/NPs/MOX-loaded scaffolds through the foam replica technique combined with the sol–gel method to improve the overall effectiveness towards bone regeneration. The structural and physicochemical characterizations were evaluated. Their mechanical properties, apatite forming ability, degradation, pharmacokinetics, and hemocompatibility were also investigated. The addition of NPs improved the compressive strength, hemocompatibility, and in vitro degradation of the composite scaffolds, resulting in them keeping a 3D porous structure and a more prolonged release profile of MOX that makes them promising for bone regeneration applications.

## 1. Introduction

Bone tissue engineering combines the principles and methods of engineering and life sciences in order to develop biological substitutes that will restore, preserve, and improve hard tissue function. This can be accomplished by integrating 3D porous scaffolds made of suitable biomaterials, cells, and biological compounds, including growth factors and active ingredients [1]. In order to reproduce the complex morphology of osteal tissue, osteoinductive materials that can integrate with the biological environment and promote bone regeneration are used in the fabrication of composite biodegradable and bioactive 3D porous scaffolds. The disadvantages of autologous grafts, as well as the prolonged healing process and elevated risk of infection, are all eliminated by the use of composite scaffolds [2].

The use of various artificial three-dimensional (3D) scaffolds for the regeneration and restoration of bone tissue has been the subject of extensive research in recent years. The biggest problem is the inability to create neovascularization in the area of bone damage, which prevents the normal homeostasis of bone tissue and leads to defective bone. The creation of scaffolds aimed at enhancing angiogenesis could be a reliable solution to the problem [3]. The restoration of bone damage is a challenge, especially in osteoporotic patients, where the loss of bone mass coexists with the degradation of the structural architecture of the bones [4].

The most important requirement for an ideal scaffold for bone tissue engineering applications is a highly interconnected porous structure with pores in the range of 200–500 μm, which is essential for bone formation, nutrient delivery, and neovascularization. Pores in the range 0–200 µm play an important role in cell adhesion, enhancing the flow of biological fluids [5].

The foam replica technique was the first scaffold synthesis method established to produce ceramic materials with controlled porosity [6] and, in 2006, it was proposed to develop scaffolds made of bioactive glass [7]. Composite scaffolds are a positive replica of a porous template that is usually a foam based on polyurethane (PU) [7]. After several coatings with a bioactive glass paste or a sol–gel solution of a glass [8], the porous template matrix is burned while the bioactive glass is heated at a high temperature. The final 3D structures own a highly open and interconnected porous network that mimics the actual architecture of the cancellous bone [7]. Silica-based nanoparticles or mesoporous particles, biodegradable polymers, and polymeric microspheres can be used to coat bioactive glass-based scaffolds developed using the foam replica technique, turning them into local drug delivery systems and enhancing their mechanical properties [9].

Bioceramic scaffolds, despite their important biological properties, exhibit limited mechanical integrity. Their fragility could be addressed by combining synthetic/biodegradable polymers. The most sought-after candidates for tissue engineering applications are biodegradable polymers with high processing flexibility [10]. In tissue engineering, synthetic biodegradable polymers with clearly defined structures and no immunological reactions are frequently used [11]. The most widely used synthetic biodegradable polymers in tissue regeneration, aliphatic polyesters, can form stable porous materials that do not dissolve or melt in vitro and are used as 3D scaffolds. The degradation of such polymers is achieved through the hydrolysis of esters [12,13]. Some of the most popular polyesters include poly(lactic acid) (PLA), polyglycolic acid (PGA), and their copolymer, poly(lactic-co-glycolic) acid (PLGA) [14].

The US Food and Drug Administration approved the synthetic copolymer of poly(lactic acid) (PLA) and polyglycolic acid (PGA) for biomedical applications. The ratio of PLA to PGA in the copolymer structure can be changed to control the degradation rate of PLGA, which can range from weeks to months [15]. Consequently, the optimum PLA/PGA ratio could give the scaffolds a desired rate of degradation and improved mechanical properties [14].

Ceramic nanocomposites constitute a biomimetic approach to bone regeneration as they mimic the structure of healthy bone and combine unique biocompatible and stimulating properties with excellent mechanical performance [16]. Silica-based nanoparticles used in biomedical applications typically have diameters between 10 and 100 nm, and their biological response is affected by their size and specific surface area [17]. These properties are also important for the application of nanobioceramics as fillers in composite materials and polymeric coatings for ceramic scaffolds [18]. Nanobioceramic–polymeric structures mimic the organic–inorganic natural structure of bone, providing nanosized crystalline materials with biomineralization capacity acting as nanoscale hydroxyapatite crystals embedded in a polymeric matrix, similar to the collagen phase of bone [19].

Although various inorganic biomaterials have been used to repair damaged tissues, silicate biomaterials, such as bioactive glass, CaSiO_3_, and Ca-Si-M ceramic systems (where M = Mg, Zn, Ti, Zr), exhibit the characteristic property of releasing Si ions in such a concentration that the growth of osteoblasts is stimulated, which makes these materials suitable for application in bone regeneration [20]. That is, glass and glass–ceramic materials containing Si, Ca, and Mg have been proven to exhibit excellent bioactivity and can be applied in biomedical applications due to improved properties relating to tissue regeneration [21]. The ternary system SiO_2_-CaO-MgO can produce different bioactive phases, such as diopside (CaMgSi_2_O_6_), akermanite (Ca_2_MgSi_2_O_7_), and bredigite (Ca_7_MgSi_4_O_16_). It has been proven (also in vivo and in vitro) that such materials are characterized by excellent bioactivity, remarkable mechanical strength [22], and their biodegradability, while the ionic derivatives of the materials mentioned above favor cell proliferation [23].

Researchers have begun to investigate the use of metal ions as therapeutic agents in the field of tissue engineering. Certain metal ions, which are also necessary for the production of enzymes, enhance bone formation, which is attributed to their stimulatory effect on the mechanism of osteogenesis (Ca and Mg) and angiogenesis (Cu). The use of metal ions, such as copper, zinc, and silver, brings about further therapeutic effects due to their anti-inflammatory and antimicrobial properties [24].

Although some ions are found in the body at trace concentrations, such as strontium, copper, zinc, and cobalt, in vitro studies with inorganic ions released from bioactive glass have shown that, although they increase ion concentration, these biomaterials exert stimulatory effects on cells and, in many cases, no other toxic effects. Copper ions stimulate angiogenesis and promote the formation and maturation of blood vessels. Sr increases osteogenesis both in vivo and in vitro, and Cu increases angiogenesis and increases the differentiation of human mesenchymal stem cells. Moreover, Cu also inhibits bone resorption [24,25].

Local delivery of active substances has advantages over oral administration for the treatment of bone infections as local antibiotics can be administered to treat bacterial adhesion during the early stages of tissue healing, thus avoiding the creation of inflammation [26]. Alternative fluoroquinolone antibiotics of the fourth generation, such as moxifloxacin (MOX), exhibit antimicrobial activity against both aerobic and anaerobic bacteria, including S. aureus, the primary pathogen linked to osteomyelitis. As moxifloxacin is effective against Staphylococcus aureus, it may be used topically to eradicate bacteria [20].

In conclusion, ion release from multifunctional ceramic-based scaffolds can create an ideal microenvironment for the formation of new bone aiming to accelerate osseointegration and promote angiogenesis. Combined with the capacity of local drug administration, this could be highly beneficial in certain dental and orthopedic applications, offering a proactive strategy for dealing with bone infections. The aim of this study was the fabrication of bioactive polymeric–ceramic composite scaffolds with prolonged and sustained MOX release using nanofillers for enhanced mechanical properties.

## 2. Materials and Methods

### 2.1. Synthesis and Characterization of Ceramic Nanoparticles

Bioactive ceramic nanoparticles (NPs) were prepared using a modified sol–gel method [27,28]. Seven different silica-based NPs were fabricated with the incorporation of different elements (Table 1). Firstly, in an ultra-pure H_2_O, ethanol, and HNO_3_ mixture, the hydrolysis of tetraethyl orthosilicate (TEOS) was performed at a molar ratio of TEOS/d.d.H_2_O/HNO_3_/ethanol = 1:12:72:10. Ca, Mg, Sr, and Cu were added as nitrate salts at room temperature. Ammonia solution was added dropwise under magnetic stirring in an ultrasonic bath. After the gelation of the solution, the prepared gel was dried at 75 °C for 2 days and then heated up to 700  °C for 3 h with a heating rate of 3  °C/min. Τetraethyl orthosilicate (TEOS), Ca(NO_3_)_2_4H_2_O, Mg(NO_3_)_2_6H_2_O, Cu(NO_3_)_2_3H_2_O, and Sr(NO_3_)_2_ were used as reactants (Sigma-Aldrich, now Merck KGaA, Darmstadt, Germany).

The physicochemical characterization of the synthesized ceramic nanoparticles was performed using Fourier-transform infrared spectroscopy (FTIR), X-ray diffraction (XRD), particle size distribution and Z-potential measurements via laser dynamic light scattering (DLS), scanning electron microscopy and energy dispersive spectroscopic analysis (SEM-EDS), transmission electron microscopy (TEM), and X-ray fluorescence spectroscopy (XRF) (see the Supplementary Materials).

For the apatite-forming ability study, all samples were immersed in an SBF solution and kept in an incubator (Incucell 55, BMT Medical Technology, Zábrdovice, Czech Republic) at 37 °C at a concentration of 1.5 mg/mL. After the initial immersion, SBF was renewed after 6 h, 24 h, and then every 48 h. The samples were removed and collected at different times and allowed to dry at room temperature [29].

To evaluate the hemolytic activity of NPs and scaffolds, whole blood was collected from healthy blood donors, after obtaining their written consent, at the Blood Donor Center of the Naoussa Hospital, which was performed under ethical approval (ID_233205920). Different concentrations of NPs (12.5, 30, 60, 125, and 250 μg/mL) received from a stock solution (1 mg/mL) were incubated with erythrocytes for 1 h and 24 h at 37 °C. As negative control (Ctrl −) and positive control (CTRL +), respectively, the supernatant of pure erythrocytes without NPs and that of erythrocytes treated with distilled water were employed. The hemoglobin released from the lysed erythrocytes was measured spectrophotometrically at 541 nm with a reference wavenumber of 700 nm [20].

### 2.2. Synthesis and Characterization of Akermanite Powder

In order to investigate the optimal synthesis conditions of the 3D bioceramic porous scaffolds, different bioactive powders were fabricated in the composition of akermanite using the sol–gel technique. In detail, TEOS, distilled H_2_O, and 2N of HNO_3_ were combined according to Wu et al. in the following molar ratio: TEOS/H_2_O/HNO_3_ = 1:8:0.16 [30]. They were stirred until the solution became clear. Calcium and magnesium were added to the solution as nitrate salts in the following molar ratio: TEOS/Ca(NO_3_)_2_4H_2_O /Mg(NO_3_)_2_6H_2_O = 2:2:1. The reactants were stirred for 5 h at room temperature until the solution became a soft gel. The solution was then transferred into a furnace at 60 °C for 2 h. Part of the composition was collected after heating to 60 and 700 °C (BC60 and BC700, respectively) and studied using the TGA/DSC technique, where, with its help, the sintering temperatures of 960, 1285, 1300 and 1400 °C were selected (BC960, BC1285, BC1300, and BC1400, respectively).

The physicochemical characterization of the synthesized powders was performed with the use of TG/DSC, FTIR, and XRD, while apatite-forming ability was also evaluated in simulated body fluid (SBF) [29]. FTIR and XRD were performed as described in the Supplementary Materials, while the apatite-forming ability test was performed as described in the Section 2.1. The thermogravimetric (TG) and differential scanning calorimetry (DSC) curves were obtained using a SETARAM SETSYS TG-DTA 16/18 instrument with heating rates of 10 °C/min from room temperature to 1350 °C under air atmosphere in order to understand the structural changes upon heating of the bioactive materials, as well as their mass loss percentage.

### 2.3. Preparation of Akermanite, Akermanite/PLGA, and Akermanite/PLGA/NPs Scaffolds

Using the foam replica technique [30,31], akermanite scaffolds were developed. The 3D porous scaffolds were created using polyurethane (PU) foam as a sacrifice template. The bioactive scaffolds were fabricated using 10 × 10 × 10 mm^3^ foam pieces. The sol–gel solution was obtained as described above. The foams were immersed in the sol–gel after the hydrolysis reaction and stirred mechanically for five minutes. The samples (green bodies) were collected and squeezed to remove the sol excess from the pores, while the green bodies were dried overnight. Sol–gel droplets were poured to adjust the thickness of the bioactive glass on the green bodies. Centrifuging the green bodies, the excess sol was removed. TGA-DSC thermal characterization of the bioceramic powder was performed to determine the sintering temperatures of the coated scaffolds, as previously reported. The temperatures chosen were 700 and 1300 °C (Ak700 and Ak1300 before and after sintering, respectively). Subsequently, the akermanite scaffolds were immersed for 3 s into 10% *w*/*v* PLGA (75:25; PLA:PGA)–acetone solution for polymer coating. After that, the scaffolds were dried at 60 °C for 2 h to completely remove the solvent. The same process was followed for the coating of akermanite scaffolds with a composite nanobioceramic–polymer coating. In order to achieve better dispersion, the nAkCuSr nanofillers were milled via planetary ball milling (S100, Retsch GmbH, Haan, Germany) for 1 h at 300 rpm using acetone as a medium. The NPs were then added to 10% *w*/*v* PLGA (75:25; PLA:PGA)–acetone solution at concentrations of 15 and 30% *w*/*v* (AkP15N and AkP30N).

### 2.4. Characterization of Akermanite, Akermanite/PLGA, and Akermanite/PLGA/NPs Scaffolds

#### 2.4.1. Assessment of Physicochemical and Mechanical Properties

In order to confirm the presence of the akermanite crystalline phase and poly(lactic-co-glycolic) acid (PLGA) due to the polymer coating on the surface of the scaffolds, FTIR spectroscopy was used. Moreover, XRD analysis, SEM/EDS measurements, and evaluation of the mechanical properties were also performed (see the Supplementary Materials).

#### 2.4.2. Ιn Vitro Studies and Biological Properties Evaluation

The apatite-forming ability in SBF, in vitro degradation, and the hemolytic activity of the scaffolds were assessed (see the Supplementary Materials).

For the apatite-forming ability study, all types of scaffolds were immersed in 75 mL SBF (using a scaffold to SBF concentration of 1.5 mg/mL) for a predetermined time (7, 14, and 28 days), during which the samples remained at 37 °C in an incubator. The SBF solution was renewed after 6 h and 24 h of immersion, and was then renewed every 2 days. The SBF solution was removed to collect the samples and then the studied samples were left for 24 h at 40 °C [12,29].

For the degradation rate of the scaffolds, 3 samples from each group were employed for each time point. The scaffolds were immersed at 37 ± 1°C in 50 mL of PBS solution (pH 7.4 ± 0.1). The vessels were then introduced into an incubator (MaxQ 4400 incubator, Thermo Fisher Scientific Inc., Waltham, MA, USA) at 37 ± 0.5 °C and continuously stirred at 90 rpm for different periods of time (7, 14, and 28 days). After the incubation, the samples were removed and heated to 100 ± 2°C to remove excess solvent. After drying, each sample was weighed every 2 h until the recorded mass changes were below 0.1%. The difference between the initial mass of the samples (W_o_) and the mass of the samples after immersion (W_t_) provides the mass loss. The % mass loss was calculated by the following equation [32]:

Mass Loss (%) = (W_o_ − W_t_)/W_o_ × 100% (1)

To investigate the hemolytic activity of the scaffolds, the same set of experiments as described in Section 2.1 was performed. Erythrocytes were incubated individually with 87.5 mg from each scaffold for the same time points and temperature as previously described for NPs. All samples were examined in triplicate.

#### 2.4.3. Assessment of Drug Loading and Release Kinetics

The scaffolds were immersed for 24 h with a scaffold mass to solution volume ratio of 5 mg/mL in a solution containing 1 and 3 mg/mL MOX to determine drug loading (DL). The suspension was removed, and the MOX-loaded scaffolds were then dried at room temperature [12]. All samples’ supernatants underwent HPLC analysis using a Shimadzu HPLC system (model LC-20AD, Tokyo, Japan).

DL% = [weight of drug in scaffolds]/[weight of scaffolds] × 100%(2)

For the in vitro release investigations employing the paddle method, a DISTEK dissolving apparatus (Distek, Evolution 2100C, North Brunswick Township, NJ, USA) equipped with an autosampler (DS Evolution 4300 North Brunswick Township, NJ, USA) was utilized (USP II method). Drug-loaded scaffolds were placed within dialysis cellulose membrane bags with a molecular weight cut-off of 12.400 and loaded into the appropriate sample holders. A total of 500 mL of a Phosphate Buffered Saline (PBS) solution (pH = 7.4) was used as the dissolution medium during the process, which was carried out at 37 °C and 100 rpm. Using a Shimadzu HPLC system, 2 mL of the aqueous solution was taken out of the release media and examined for the presence of moxifloxacin. The temperature of the column was 25 °C (Athena C18, 120 A, 5 m, 250 mm, 4.6 mm, CNW Technologies). Orthophosphoric acid and triethylamine were used to bring the pH of the mobile phase down to 2.57, which contained MeOH/H_2_O in a ratio of 55/45 *v*/*v*. Concentration was calculated at 293 nm using an HPLC-UV system and a previously constructed calibration curve, with 10 L of injection inside. The calibration curve was produced by diluting a stock aqueous solution of 200 ppm moxifloxacin using mobile phase to concentrations of 0.025, 0.05, 0.1, 0.5, 1, 2, 5 10, 25, and 100.

### 2.5. Statistical Analysis

The average and standard deviation (SD) of at least three separate replicated experimental procedures are used to represent each result in hemocompatibility assay. The paired independent t-test was used for the statistical analysis. The *p*-value for statistical significance was set at 0.05. Untreated cells served as negative control (N. control), while different letters suggest statistically significant differences among wells with different concentrations of NPs.

## 3. Results

### 3.1. Ceramic Nanoparticles

#### 3.1.1. X-ray Diffraction (XRD)

**Figure 1:**
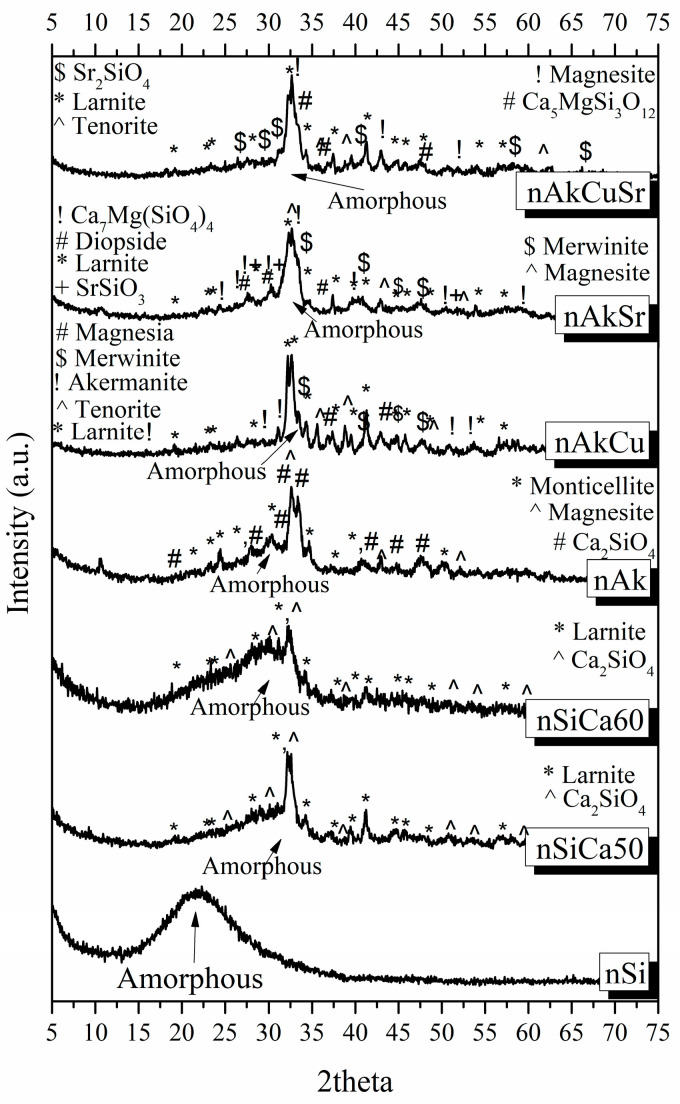
XRD patterns of the synthesized NPs.

All nanobioceramics were characterized by XRD to determine the developed crystalline phases. The results are summarized in Figure 1, while quantitative analysis of the crystalline phases is presented in Table 2. The XRD patterns showed that all NPs consisted of a high percentage of amorphous phase. More specifically, except for the nanoceramics of the nSi sample that consisted of 100% amorphous phase (glass), the percentage of the amorphous phase ranged from 36 to 75.0%, with the maximum percentage found in the nSiCa60 sample and the lowest percentage found in the nAkCuSr sample.

In the XRD patterns of all samples, the presence of calcium silicate crystalline phases was observed. In particular, the XRD patterns of the SiO_2_CaO system corresponding to the nSiCa50 and nSiCa60 samples revealed the presence of a high percentage of amorphous phase, the formation of larnite, and other calcium silicate crystalline phases, i.e., Ca_2_SiO_4_ and CaSiO_3_ for nSiCa50 and nSiCa60, respectively.

The patterns of nAk presented 49% amorphous phase, a large percentage of calcium silicate crystalline phase (25% Ca_2_SiO_4_), a smaller percentage of monticellite (15%), and a slightly smaller percentage of magnesite (11% MgCO_3_). Monticellite is a crystalline phase of calcium magnesium silicate (CaMgSiO_4_), the presence of which indicates partly the incorporation of calcium and magnesium simultaneously in the silicate matrix.

In the patterns of the nAkCu sample, 38% amorphous phase was observed, while the percentages of the crystalline phases were divided into calcium magnesium silicate crystalline phases (4% akermanite Ca_2_Mg(Si_2_O_7_) and 12% merwinite Ca_3_Mg(SiO_4_)_2_), 41% larnite, 3% magnesia (MgO), and 2% tenorite (CuO). Although crystalline calcium magnesium silicate phases are present, as well as a calcium silicate phase (larnite), the presence of magnesium oxide (magnesia) indicates that magnesium was not fully incorporated into the silica matrix. Moreover, the presence of copper oxide (CuO) without the presence of another Cu-containing crystalline phase indicates the absence of copper incorporation into the silica network.

Quantitative analysis of the nAkSr sample showed the presence of 43% amorphous phase; calcium magnesium silicate phases divided into 13% Ca_7_Mg(SiO_4_)_4_, 12% merwinite, and 4% diopside (MgCaSi_2_O_6_); and 11% larnite, 14% magnesite (MgCO_3_), and 3% SrSiO_3_. The quantitative analysis of the crystalline phases indicates the partial incorporation of calcium with magnesium simultaneously in the silica network. Accordingly, the incorporation of strontium into the silica network was observed partially.

Finally, in the nAkCuSr sample, 36% amorphous phase was observed, while the percentage of the crystalline phases was 23% Ca_5_MgSi_3_O_12_, 26% larnite, 11% magnesite, 2% Sr_2_SiO_4_, and 2% tenorite. The presence of a high percentage of calcium magnesium silicate crystalline phase indicates the incorporation of calcium and magnesium simultaneously in the silica network. In addition, the presence of the crystalline phase of Sr_2_SiO_4_ indicates the incorporation of strontium into the silicate lattice, while the appearance of copper oxide (tenorite) possibly indicates the absence of complete incorporation of copper into the lattice.

#### 3.1.2. Fourier-Transform Infrared Spectroscopy

**Figure 2:**
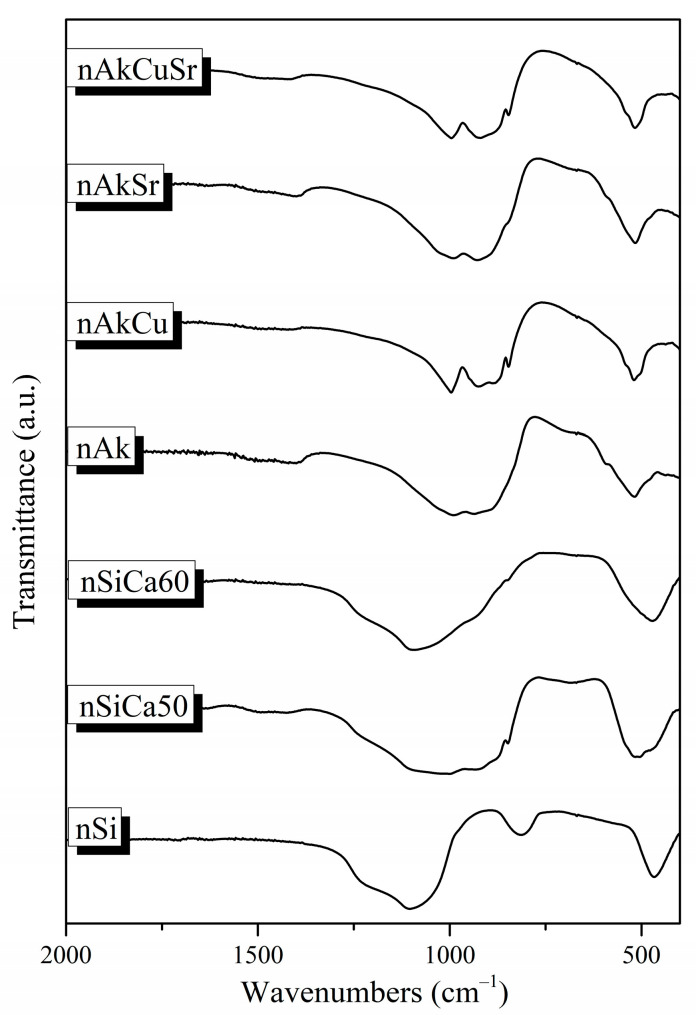
FTIR spectra of the synthesized nanobioceramics.

The FTIR spectra of the synthesized nanobioceramics presented the characteristic peaks of silicate glasses (Figure 2). More specifically, the asymmetric stretching vibration of the Si–O–Si bond is responsible for the broad peak at around 900–1200 cm^−1^, and the Si–O–Si bending vibration is responsible for the peak at about 470 cm^−1^ [20,27]. The weak peak at around 1645 cm^−1^ is attributed to the presence of adsorbed water on the surface of the samples, and the stretching vibration of the C–O bond was observed at 1410–1510 cm^−1^ on the surface of all samples, except nSi and nSiCa60 [20,27]. In addition, the spectrum of the nSi sample presented a peak around 816 cm^−1^ corresponding to the symmetric stretching of the Si–O–Si bond, while the spectra of the rest of the samples showed a shoulder around 900 cm^−1^ due to the vibration of the Si–O–Ca bond. Additionally, a lower intensity shoulder in the spectra of nAk, nAkCu, nAkSr, and nAkCuSr samples around 690 cm^−1^ is attributed to the vibration of the Si–O–Mg bond [20,27].

More specifically, in the spectra of nSiCa50 and nSiCa60 samples, the peaks at 518 cm^−1^, 849 cm^−1^, 873 cm^−1^, 924 cm^−1^, and 995 cm^−1^ can be attributed to the presence of calcium silicates, and more specifically to the formation of larnite (Ca_2_SiO_4_). Larnite formation was also observed in the spectra of nAkCu, nAkSr, and nAkCuSr samples. In the spectrum of the nAk sample, the presence of the peak at 690 cm^−1^ is attributed to the vibration of the Si–O–Mg bond and the peaks at 478 cm^−1^, 592 cm^−1^, 890 cm^−1^, 934 cm^−1^, and at 992 cm^−1^ occur in crystalline phases of calcium magnesium silicates, more specifically in the formation of monticellite (CaMgSiO_4_). Furthermore, the peaks at 518 cm^−1^, 690 cm^−1^, and 1495 cm^−1^ could be attributed to the formation of magnesium carbonates such as magnesite (MgCO_3_) [33,34]. In the spectrum of nAkCu, in addition to the peaks attributed to larnite, the presence of peaks at 522 cm^−1^, 540 cm^−1^, 880 cm^−1^, and 930 cm^−1^ are attributed to the formation of merwinite (Ca_3_Mg(SiO_4_)_2_), while the peak at 930 cm^−1^ in combination with the peaks at 848 cm^−1^ and 980 cm^−1^ could also be attributed to the presence of akermanite (Ca_2_Mg(Si_2_O_7_) [35]. In the spectrum of the nAkSr sample, in addition to the peaks that could be attributed to larnite, merwinite, and magnesite, the peaks at 477 cm^−1^, 515 cm^−1^, 682 cm^−1^, 846 cm^−1^, and 888 cm^−1^ could be attributed to the formation of diopside (MgCaSi_2_O_6_) [36]. In the spectrum of the nAkCuSr sample, in addition to the peaks that could be attributed to larnite and magnesite, the peaks at 518 cm^−1^, 542 cm^−1^, 846 cm^−1^, 876 cm^−1^, 920 cm^−1^, and 998 cm^−1^ can be observed in the spectra of crystalline phases of calcium magnesium silicates. Moreover, in the spectra of Cu-containing NPs (nAKCu and nAkCuSr), the peaks attributed to copper oxide (tenorite) were not noticed because they cannot be observed due to appearing at lower wavenumbers than 400 cm^−1^ [36].

#### 3.1.3. Particle Size Distribution and Z-Potential Measurements by Laser Dynamic Light Scattering (DLS)

The results of Z-potential measurements for all NPs are presented in Table 3. All NPs exhibit a negative ζ-potential, with values ranging from −19.3 ± 0.4 to −3.51 ± 0.4. The nAkCuSr sample presented the highest charge value.

#### 3.1.4. Scanning Electron Microscopy and Energy Dispersive Spectroscopic Analysis (SEM-EDS)

**Figure 3:**
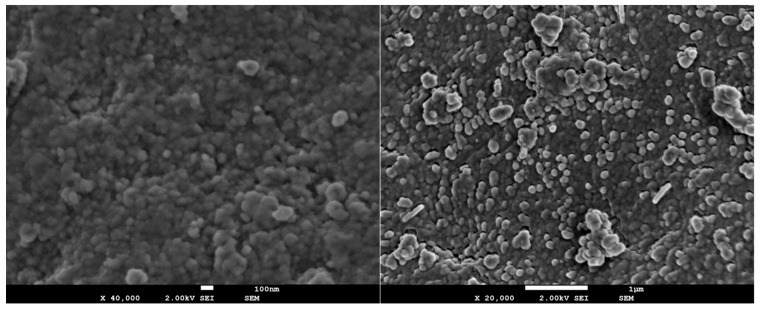
Representative SEM micrographs at ×40,000 (**left**) and ×20,000 (**right**) magnification of NPs in the composition of akermanite enriched with Cu and Sr ions (nAkCuSr).

The SEM micrographs of the synthesized nanobioceramics before immersion in SBF presented the formation of nano materials with mean particle sizes in the range of 30–120 nm, without particular differences being observable in terms of their size and dispersion (Figure 3). The smallest particle size was observed in the nAkCuSr sample, while the largest was observed in the nAk sample.

In addition, according to XRD results, samples of nAkCu and nAkCuSr, which contain copper oxide in their composition, appear to display a morphology of long narrow particles by areas which can be attributed to the presence of tenorite (copper oxide).

#### 3.1.5. Transmission Electron Microscopy (TEM)

**Figure 4:**
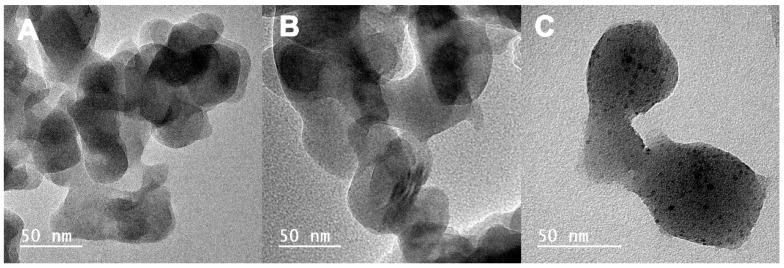
TEM micrographs of calcium magnesium silicate Ak powder samples: (**A**) Ak-neat, (**B**) AK Sr-doped, and (**C**) Ak Sr and Cu co-doped.

TEM nanographs of the Ak-based samples are shown in Figure 4. As can be seen, the nanoparticles have oval shapes with sizes less than 100 nm. The morphology of all Ak-based samples remains the same; however, agglomeration of the powder particles is also observed. Sr-doped Ak shows no differences to Ak-neat. On Sr- and Cu-doped Ak, spherical particles are present, with an average diameter of 3.5 ± 1 nm, which is likely attributed to the presence of CuO [37].

#### 3.1.6. X-ray Fluorescence Spectroscopy (XRF)

Table 4 displays the XRF-detected chemical composition of all synthesized NPs. The nominal composition was compared to the detected composition in mol% amounts, which presented high ion incorporation, especially for calcium.

#### 3.1.7. Apatite-Forming Ability

**Figure 5:**
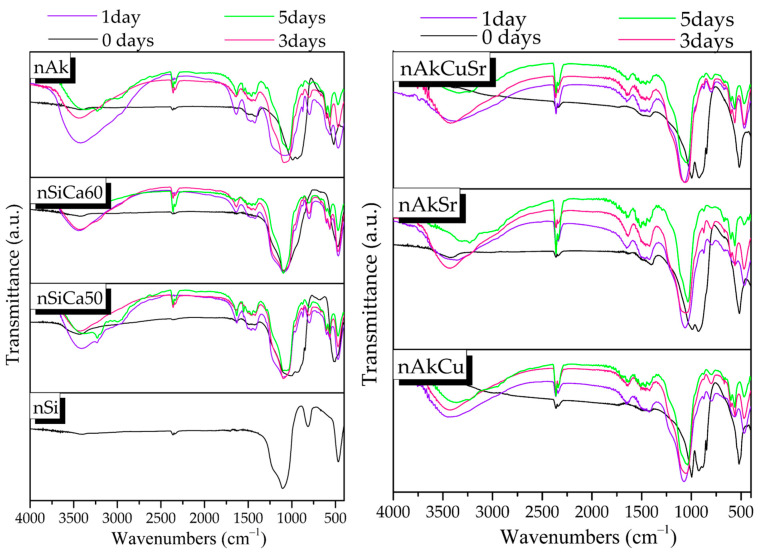
FTIR spectra of the synthesized NPs after 1, 3, and 5 days of immersion in SBF.

After 24 h of immersion in SBF, a sharpening of the broad peak around 900–1200 cm^−1^ is observed, which is attributed to the bending vibration of the phosphate group (PO_4_)^−3^. A reduction in the silicate peak at 470 cm^−1^ and a simultaneous shift to lower wavenumbers is also observed. In addition, the appearance of a weak peak around 560 to 640 cm^−1^ is assigned to the P–O bending vibration of the formed amorphous calcium phosphate phase on the surface of the nAkCu, nAkSr, and nAkCuSr samples. However, in the spectra of the nSiCa50, nSiCa60, and nAk samples, a double peak around 560 to 640 cm^−1^ is observed after the first day of immersion in SBF, which is corresponds to P–O bending vibrations (Figure 5). Finally, there is an increase in the peak around 1410–1510 cm^−1^, which corresponds to the stretching vibration of the oxygen–carbon bond, while the slight decrease in the silicate peak at 470 cm^−1^ indicates the formation of a hydroxycarbonate apatite (HCAp) layer on the surface of the nSiCa50, nSiCa60, and nAk samples (Figure 5) [28]. However, after 24 h, a peak at 750–850 cm is observed, which was not observed in any material prior to immersion in SBF. The presence of this peak corresponds to the symmetric stretching vibration of oxygen with silicon (O–Si), and since it is not observed in the samples before the immersion, it can be attributed to the formation of a layer of silanols resulting from ion exchanges that take place during the immersion of the samples in SBF. Polycondensation of silanols can be represented by the following [27]:

Si-OH + Si-OH → Si-O-Si(3)

After 3 days of immersion in SBF, a further sharpening of the broad peak at 900–1200 cm^−1^ is observed, which, as mentioned above, is attributed to the bending vibration of the phosphate group (PO_4_)^−3^. Furthermore, a peak at 960 cm^−1^ is observed, which corresponds to the stretching vibration of the phosphate group (PO_4_)^−3^; the formation of a double peak around 560–640 cm^−1^ is observed, which is attributed to the bending vibration of phosphate groups (PO_4_)^−3^; and a further increase in the peak around 1410–1510 cm^−1^, which corresponds to the stretching vibration of the oxygen–carbon bond, along with a slight decrease in the silicate peak at 470 cm^−1^ indicates the formation of a hydroxycarbonate apatite (HCAp) layer on the surface of the nAkCu, nAkSr, and nAkCuSr samples (Figure 5) [28]. After 5 days of immersion in the SBF, a further sharpening of the peaks attributed to apatite was observed. Additionally, the peak at 750–850 cm^−1^ in the spectra of the samples after 24 h of immersion associated with the stretching vibration of oxygen–silicon bonds and a decrease in the peak at 470 cm^−1^ are attributed to the further growth of the hydroxycarbonate apatite layer.

#### 3.1.8. Hemolysis Assay

**Figure 6:**
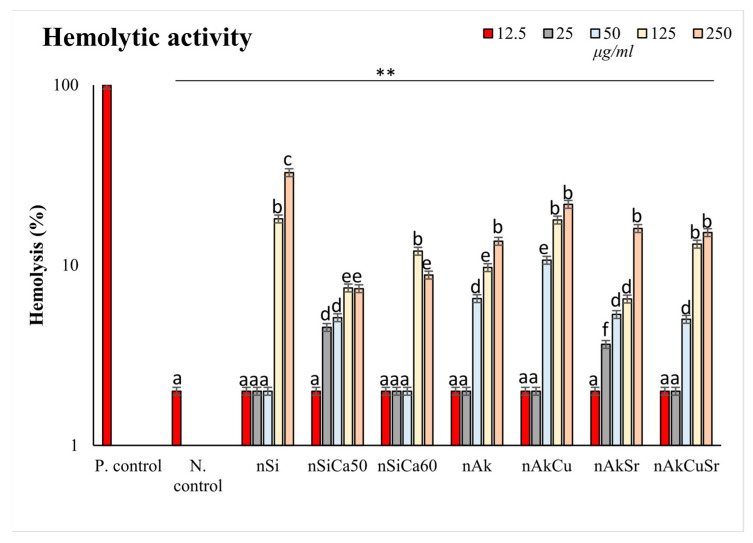
Hemocompatibility of silica-based NPs at different concentrations (12.5, 25, 50, 125, and 250 μg/mL) at body temperature (37 °C) after 24 h of incubation. ** indicates statistically significant differences (*p* < 0.001) among negative control (N. control) and cells incubated with NPs, while different letters suggest statistically significant differences among concentrations.

Ion-doped silica-based nanoparticles (NPs) were exposed directly to healthy human erythrocytes for 24 h, and their hemocompatibility was subsequently examined. Figure 6 shows the hemolytic activity of the newly synthesized NPs at body temperature (37 °C). None of the silica-based NPs induced hemolysis at concentrations below 25 μg/ml. Undoped NPs presented statistically significant higher (*p* < 0.001) hemolysis (approximately 8–20%) at 125 and 250 μg/mL compared with all doped NPs tested. In the case of NPs doped with 40% calcium (nSiCa60), increased hemocompatibility was recorded, which is consistent with previous studies by our group [38]. Akermanite (calcium and magnesium-doped silica) NPs (nAk) also exhibited an optimum hemodynamic profile, although the behavior was also dose-dependent (*p* < 0.05). Silica-based NPs doped with calcium, magnesium, and copper (nAkCu) showed the second-least hemocompatible profile with respect to dosage compared with all tested NPs. On the other hand, NPs doped additionally with strontium alone (nAkSr) and in combination with copper (nAkCuSr) showed better hemocompatibility, making this combination the most suitable for future studies. The average NPs concentration that induced 12% hemolysis was 125 μg/mL. The hemocompatible concentration for all NPs was 12.5 μg/mL.

### 3.2. Akermanite Powder

#### 3.2.1. Thermogravimetric Analysis—Differential Scanning Calorimetry (TGA-DSC)

**Figure 7:**
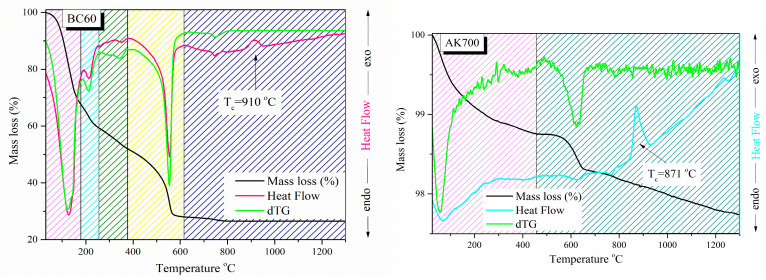
Heat flow and mass loss curves of samples after heating to 60 and 700 °C (BC60 and BC700, respectively).

Samples of the initial powder were taken and studied using the TGA-DSC technique after thermal treatment at 60 °C and 700 °C (samples BC60 and BC700, respectively) to determine the optimum sintering temperature of the akermanite-based ceramic scaffolds. Heat flow and mass loss curves were obtained, as shown in Figure 7.

Both samples’ heat flow curves displayed endothermic broad peaks at low temperatures (below 200 °C), which are attributed to the processes of water evaporation (adsorbed water and water produced during the polycondensation reaction of silanol) and HNO_3_ evaporation [39]. Secondary endothermic peaks of smaller range and intensity were observed in sample BC60 up to a temperature of 400 °C, and one of clearly higher intensity was observed in the range of 550 to 600 °C, probably due to the thermal decomposition of nitrate compounds [39] Both samples also showed endothermic peaks of low intensity, which are possibly due to short-range decay phenomena of certain phases. Exothermic peaks in the range of 850–950 °C were also observed in the heat flow curves of both samples, which, according to Myat Myat-Htun et al., can be attributed to the formation of crystalline akermanite [40]. The crystallization temperature of each sample is presented in Figure 7 and Table 5. According to these results, a shift in the crystallization temperature to lower temperatures is observed with an increase in the initial heating of the studied samples.

The TG curves indicate that the mass loss up to 600 °C is quite extensive for sample BC60 (~76%) and more limited for the BC700 sample. This fact is due to the low heating temperature of the material before the thermal profile studies (60 °C). Mass loss in the range of 600 °C to the crystallization temperature is insignificant for both samples. Furthermore, it was observed that a further increase in temperature after the crystallization temperature leads to limited mass loss. Based on the TGA-DSC results and considering that the formation of akermanite is possible for temperatures higher than Tc, sintering temperatures of 960, 1300, and 1400 °C (BC960, BC1300, and BC1400, respectively) were chosen to investigate the optimum temperature for akermanite formation while seeking to maintain the three-dimensional porous structure.

#### 3.2.2. Fourier-Transform Infrared Spectroscopy

**Figure 8:**
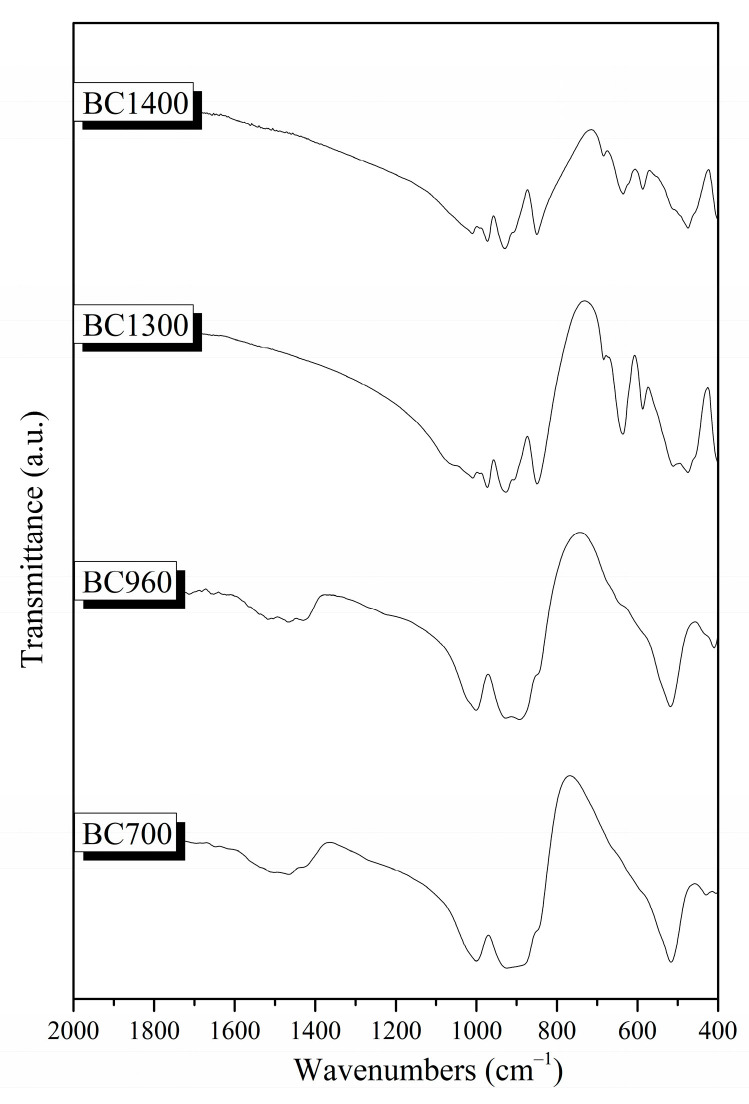
FTIR spectra of the synthesized bioceramic powders.

Figure 8 shows the FTIR spectra of the synthesized powders sintered at different temperatures (700, 960, 1300, and 1400 °C, respectively). All samples showed the characteristic peaks of silicate glasses described in Section 3.1.2 [20,27,28,38,41]. The C–O bond’s stretching vibration was observed in the region of 1410–1510 cm^−1^ on the surface of lower-temperature heat-treated samples (samples BC700 and BC960). Furthermore, the shoulder at around 900 cm^−1^ corresponds to the vibration of the Si–O–Ca bond [20,27,38,41]. The presence of the peak around 690 cm^−1^ in the spectra of the BC1300 and BC1400 samples, and the low-intensity shoulder in the spectra of the BC700 and BC960 samples, is attributed to the vibration of the Si–O–Mg bond [42].

More specifically, the presence of peaks around 520 cm^−1^, 540 cm^−1^, 880 cm^−1^, and 930 cm^−1^ in the spectra of all samples is attributed to the formation of merwinite (Ca_3_Mg(SiO_4_)_2_). Moreover, in the spectra of the BC1300 and BC1400 samples, the peak at 930 cm^−1^, along with the peaks at 472, 588, 636, 684, 850, 905, and 975 cm^−1^, could be attributed to the presence of akermanite (Ca_2_Mg(Si_2_O_7_)) [35]. Finally, in the BC1300 sample, the peaks at 476 cm^−1^, 514 cm^−1^, 684 cm^−1^, and 848 cm^−1^ could be attributed to the formation of diopside (MgCaSi_2_O_6_) [35].

#### 3.2.3. X-ray Diffraction (XRD)

**Figure 9:**
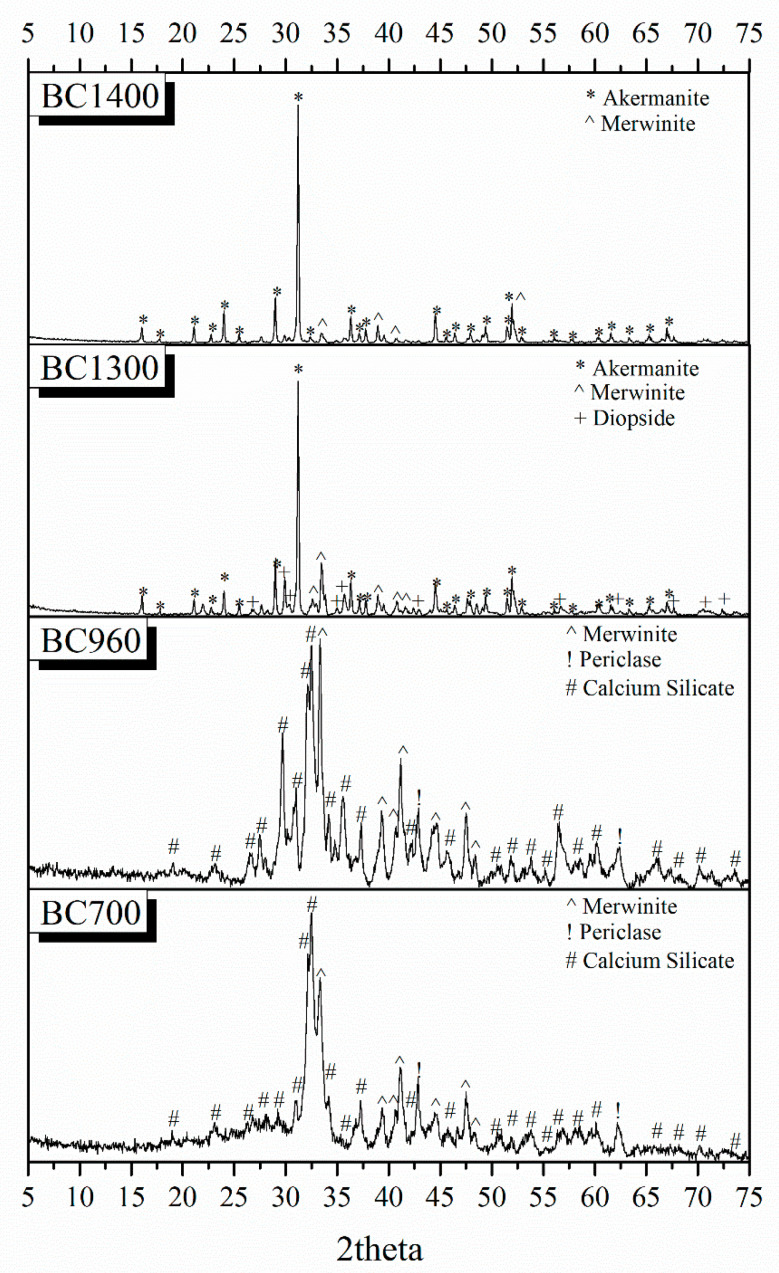
XRD patterns of the bioceramic powders.

Figure 9 presents the X-ray diffraction patterns of the nanobioceramic powders investigated to study the formation of crystalline phases after heat treatment.

The quantitative analysis of the XRD patterns is presented in Table 6. All bioactive powders consist of a low to zero amount of an amorphous phase. More specifically, the BC700 sample consists of 14% amorphous phase, 66% crystalline calcium silicate phase (Ca_2_SiO_4_), 17% merwinite (Ca_3_Mg(SiO_4_)_2_), and 3% periclase (MgO). BC960 consists of a smaller percentage of amorphous phase (8%), 54% crystalline calcium silicate phase (Ca_2_SiO_4_), 35% merwinite (Ca_3_Mg(SiO_4_)_2_), and 3% periclase (MgO). The presence of magnesium oxide (periclase) possibly indicates the inability to fully incorporate magnesium into the lattice at temperatures below 960 °C. The BC1300 and BC1400 samples consist of a high percentage of akermanite, which increases with increasing temperature. More specifically, quantitative analysis of the XRD patterns of sample BC1300 shows that it consists of 90% akermanite, 7% merwinite, and 3% diopside. Finally, as expected, sample BC1400 consists mainly of akermanite (96%) and a small percentage of merwinite (about 4%).

#### 3.2.4. Apatite-Forming Ability

**Figure 10:**
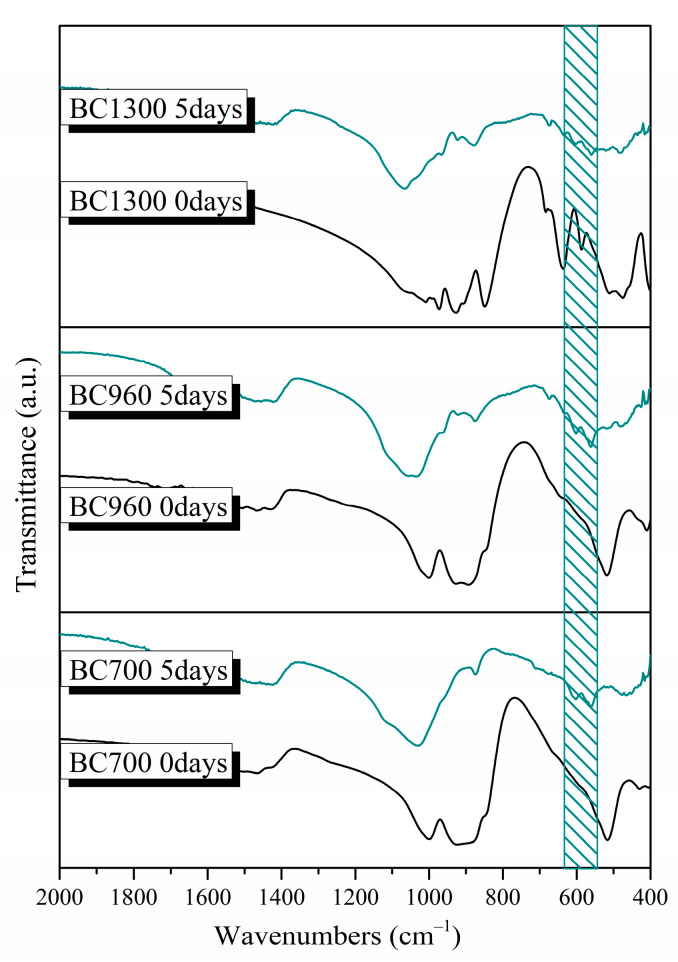
FTIR spectra of the bioceramic powders after 5 days of immersion in SBF. The shadow area between 560 and 640 cm^−1^ highlights the double peak attributed to the formation of an apatite layer on the surface of all samples.

After 5 days of immersion in SBF, a sharpening of the broad peak between 900 and 1200 cm^−1^ is observed due to the bending vibration of the phosphate group (PO_4_)^−3^. The reduction in the peak at 470 cm^−1^ (Si-O-Si bending vibration) and its shift to lower wavelengths, along with the simultaneous presence of a double peak around 560 to 640 cm^−1^, indicates the formation of an apatite layer on the surface of all samples (Figure 10). Additionally, the peak at around 960 cm^−1^, which is not observed in the spectra of the samples before immersion in SBF, corresponds to the stretching vibration of the phosphate group (PO_4_)^−3^. Finally, in the spectra of sample BC1300, the presence of the peaks around 1410–1510 and 870 cm^−1^, which are not observed in the original sample, corresponds to the stretching vibration of the C-O bond. The presence of these peaks, in combination with a small reduction in the peak at 470 cm^−1^, indicates the development of a hydroxyapatite carbonate (HCA) layer on the surface of the samples [27,38].

### 3.3. Akermanite-Based Scaffolds

#### 3.3.1. Fourier-Transform Infrared Spectroscopy

**Figure 11:**
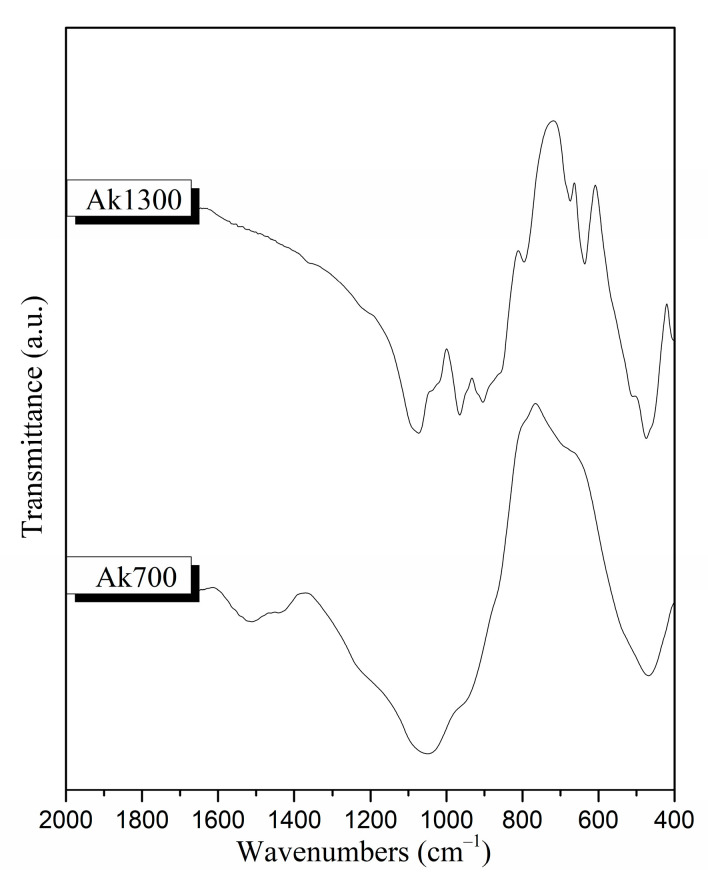
FTIR spectra of scaffolds after being heated to 700 and 1300 °C (Ak700 and Ak1300, respectively) before immersion in c-SBF.

The FTIR spectra for the ceramic scaffolds before moxifloxacin encapsulation and polymeric coating are presented in Figure 11. Both samples before and after the sintering process (sample Ak700 and Ak1300, respectively) show the characteristic peaks of silicate glasses described in Section 3.1.2 [20]. In the region of 1410–1510 cm^−1^, the stretching vibration of the C–O bond is observed in the spectra of the scaffolds before sintering (sample Ak700). Moreover, at around 690 cm^−1^, a peak for the Ak1300 sample and a shoulder for the Ak700 sample are attributed to the vibration of the Si–O–Mg bond [20]. Furthermore, the shoulder at 550 cm^−1^ is attributed to the bending vibration of the Ca–O bond [43].

Furthermore, the presence of shoulders at approximately 516 cm^−1^, 540 cm^−1^, and 880 cm^−1^ in the spectrum of sample Ak1300 is attributed to merwinite formation. In the spectrum of Ak1300, the peaks at 474, 638, 676, 854, 908, and 968 cm^−1^ could be attributed to the presence of akermanite [35]. Finally, in the spectra of the Ak1300 sample, the peaks at 474 cm^−1^, 676 cm^−1^, and 854 cm^−1^ that were attributed to the presence of akermanite could, in combination with the peak at 512 cm^−1^, be attributed to the formation of diopside (MgCaSi_2_O_6_) [35]. The absence of peaks that are attributed to crystalline phases in the spectrum of the Ak700 sample is due to the low heating temperature. Sample Ak1300 will hereafter be referred to as Ak.

**Figure 12:**
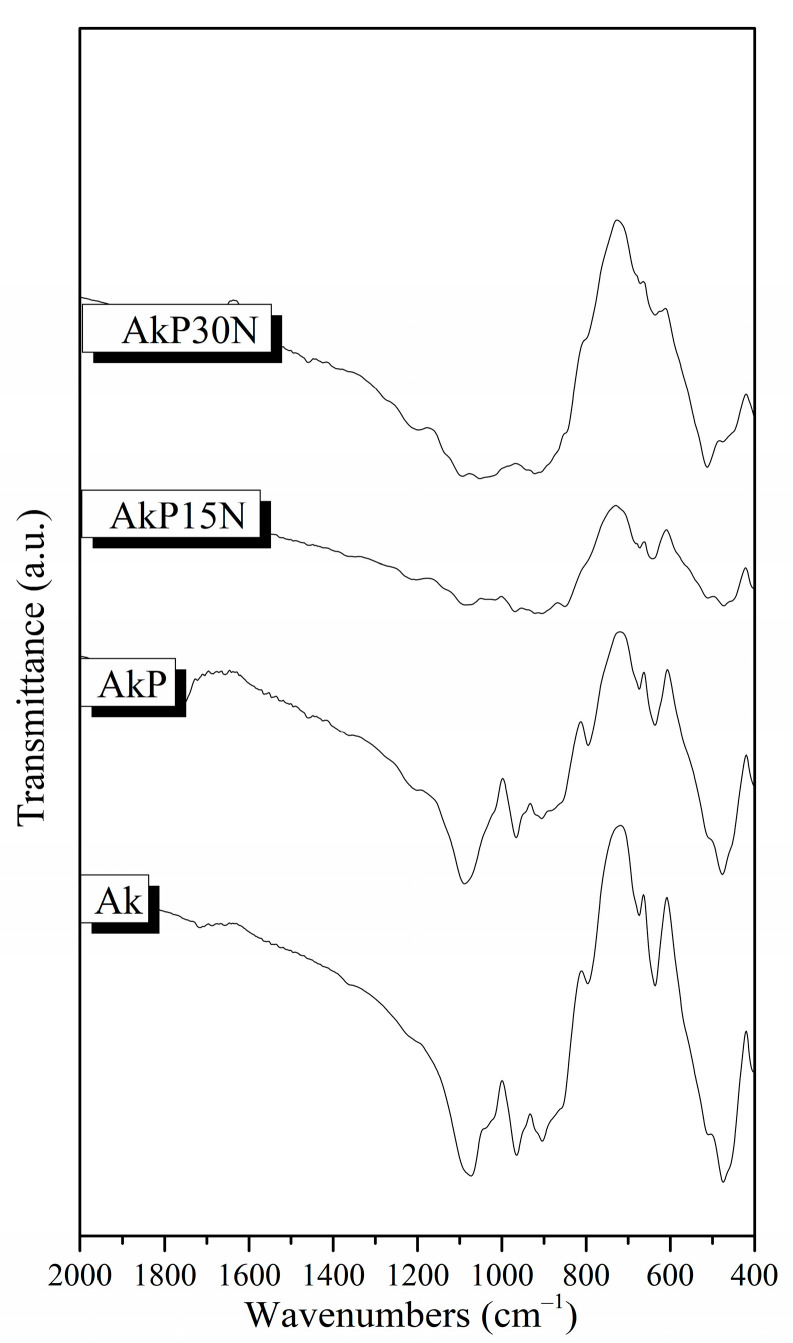
FTIR spectra of synthesized scaffolds before immersion in SBF.

The FTIR spectra of the PLGA-coated akermanite scaffolds with the addition of 0, 15, and 30% *w*/*v* nanoparticles of the nAkCuSr system (AkP, AkP15N, and AkP30N, respectively) are presented in Figure 12. All PLGA-coated samples showed the characteristic peaks that are attributed to PLGA. More specifically, the peaks at 1182 and 1089 cm^−1^ (C–O–C stretch), 1752 cm^−1^ (C=O carbonyl stretch), and 1454 cm^−1^ (C–H bond stretch) were observed in all samples [35]. The shoulder at 1050 cm^−1^ in all samples is attributed to the C–CH_3_ vibration, and the peak at 1268 cm^−1^ in the spectra of the AkP15N and AkP30N samples is attributed to the ester groups of PLGA [35].

In the spectra of samples AkP15N and AkP30N, the peaks observed at 518 cm^−1^, 846 cm^−1^, and 920 cm^−1^ are attributed to crystalline phases of magnesium-, calcium-, and silicon-based materials, such as larnite and magnesite. The presence of these peaks only in samples containing 15 and 30% *w*/*v* nAkCuSr nanoparticles within the PLGA polymeric matrix (AkP15N and AkP30N, respectively) can be attributed to the presence of nAkCuSr nanoparticles.

#### 3.3.2. Scanning Electron Microscopy and Energy Dispersive Spectroscopic Analysis (SEM-EDS)

**Figure 13:**
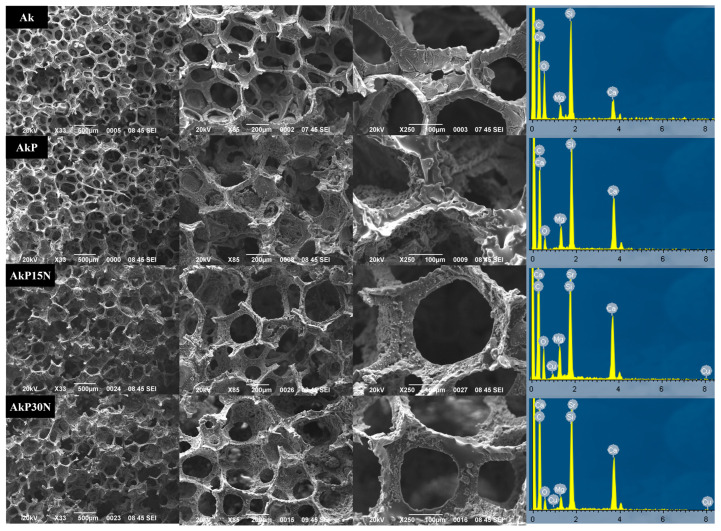
SEM micrographs and the EDS spectra of all types of scaffolds.

Figure 13 show representative SEM micrographs of each group of scaffolds. All scaffolds appear to display a fine porous structure, although in some cases some pores appear to be partially closed. This fact, however, does not seem to reduce the interconnectivity inside the scaffolds.

**Figure 14:**
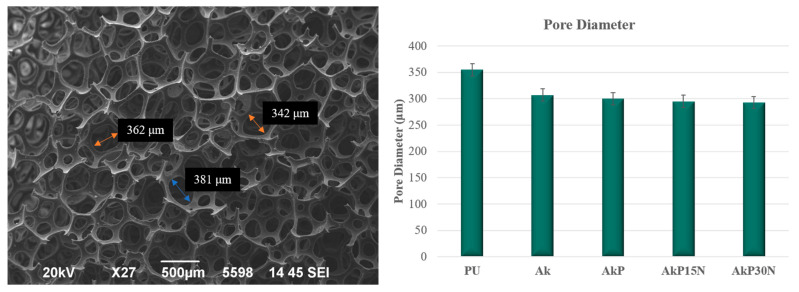
SEM micrograph of polyurethane foam (**left**) and the determination of the average pore size of initial polyurethane foam and synthesized scaffolds (**right**).

SEM images of the polyurethane (PU) foam were taken to determine its average pore size prior to the coating process with the colloidal gel solution. As shown in Figure 14, the pore size for all groups of scaffolds ranged from 293 to 307 μm.

The SEM images after PLGA co-polymer coating did not demonstrate closure of the pores of the akermanite scaffolds (AkP group), and the scaffolds showed a uniform macroporous structure with high interconnectivity, which is essential for bone tissue engineering applications. The struts of this group are also characterized by microparticles and nanopores, which act as the drug loading matrix [35]. The 10% PLGA coating partially filled the micropores and nanopores or the struts while maintaining the high interconnectivity of the macropores. This was also observed after the addition of 15 and 30% nanoparticles of the nAkCuSr sample to the PLGA polymeric matrix, forming a relatively smooth surface on the coated scaffold surfaces. As can be seen in Figure 13 with the AkP15N and AkP30N groups, the presence of nanoparticles did not negatively affect the size of the pores or the morphology of the scaffolds; on the contrary, their uniform dispersion is observed on the surface of the samples. Moreover, the presence of the NPs within the polymeric matrix is also confirmed by the presence of the additional peaks of Cu and Sr in the EDS spectra of the scaffolds containing nanoparticles (the AkP15N and AkP30N groups).

#### 3.3.3. Compressive Strength Evaluation

**Figure 15:**
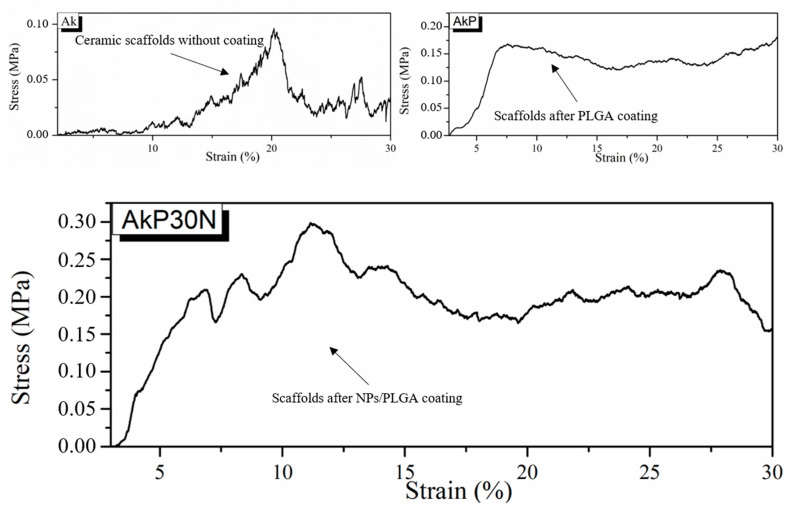
Indicative stress—strain (%) curves of the developed scaffolds.

Evaluation of the mechanical strength of all groups of scaffolds under compression load revealed that bioceramic scaffolds presented limited mechanical stability. Figure 15 presents the typical stress and strain curves of all groups, confirming the brittle nature of bioceramics. The continuous peaks of absorbed stress before the layer-by-layer fracture of the pore walls and struts, which can be seen as valleys in the curves, indicate the fragility of the bioceramic scaffolds (Ak). However, it seems that the PLGA and NPs/PLGA-coated scaffolds presented better stress performance, probably due to effective infiltration of the coating within the micropores of the struts. The average mechanical strength of scaffolds under compression is shown in Figure 15.

**Figure 16:**
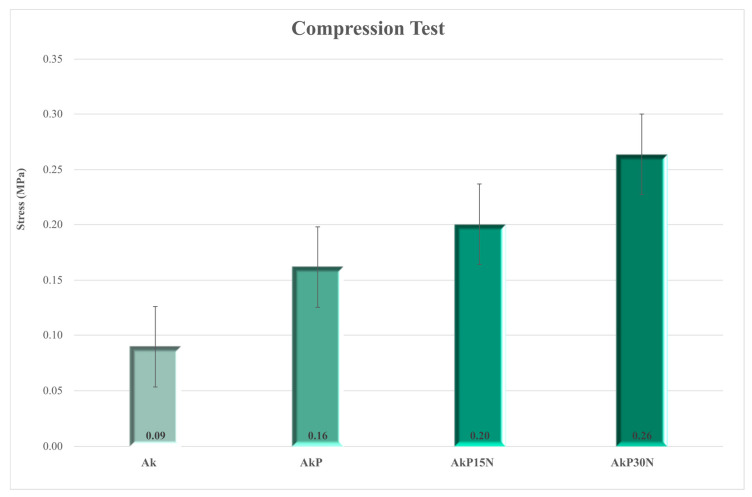
Mean compressive strength of synthesized scaffolds.

More specifically, the scaffolds of the Ak group, which correspond to the akermanite scaffolds before being coated with the PLGA polymer, presented limited mechanical strength (mean compressive strength of 0.09 MPa). The relatively low average stress value of akermanite scaffolds does not make them suitable for applications involving high load stress, though they do have the necessary strength to withstand handling procedures during implantation. Although the mechanical strength of the akermanite scaffolds before being coated with the PLGA polymer was limited, a slight enhancement was observed with the contribution of the polymer. The scaffolds of the AkP group, which correspond to the akermanite scaffolds after PLGA coating, showed a mean compressive strength of 0.16 MPa. The mechanical strength results of the AkP15N and AkP30N scaffolds, which correspond to akermanite scaffolds coated with 10% *w*/*v* PLGA and 15 or 30% *w*/*v* nanoparticles from the nAkCuSr group, respectively, presented clearly enhanced mechanical strength. The average value of mean compressive strength, as shown in Figure 16, reached 0.20 and 0.26 MPa for the AkP15N and AkP30N groups, respectively, suggesting that increasing the percentage of nanobioceramics within the polymer coating increases the mechanical strength of the scaffolds.

#### 3.3.4. X-ray Diffraction (XRD)

**Figure 17:**
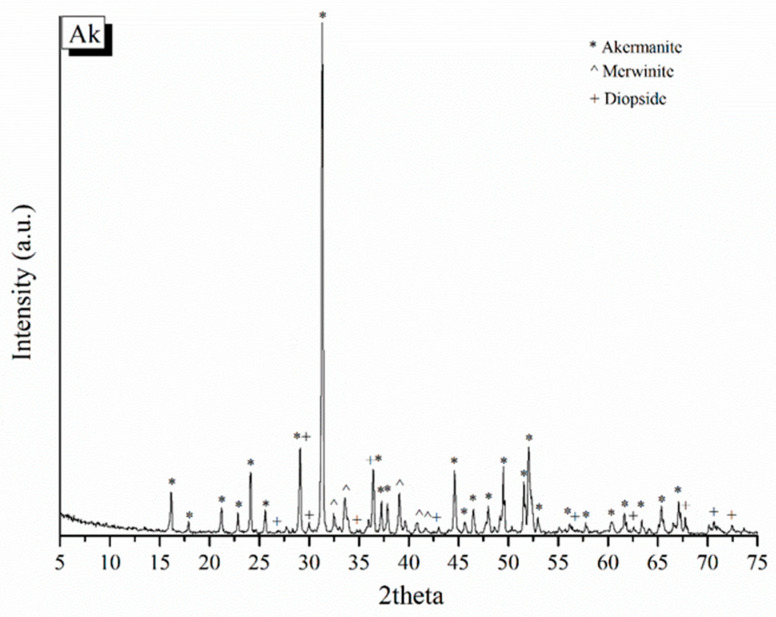
X-ray patterns of akermanite scaffolds.

The X-ray diffraction results of the bioceramic scaffolds (Ak) were studied to determine the crystalline phases formed (Figure 17). The quantification of the crystalline phases is presented in Table 7. More specifically, Ak scaffolds consist of 100% crystalline phases, which are divided into 93% akermanite, 4% merwinite, and 3% diopside. Meanwhile, the quantification results of the crystalline phases of the akermanite scaffolds (Ak) did not differ much from those of the bioceramic powder BC1300, which was reproduced in a three-dimensional porous structure following the same thermal steps.

#### 3.3.5. In Vitro Degradation

**Figure 18:**
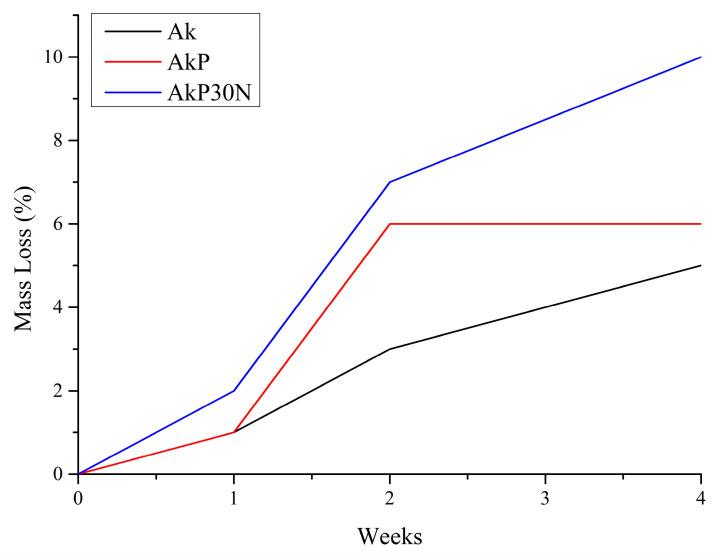
Results of in vitro degradation evaluation of the synthesized scaffolds.

The in vitro degradation study of all groups of scaffolds is presented in Figure 18 and Table 8. More specifically, the akermanite (Ak) scaffolds showed increasing solubility, presenting an average mass loss of 1.1, 3.4, and 5.4% wt after 1, 2, and 4 weeks of incubation, respectively. PLGA-coated akermanite scaffolds (AkP) showed 1.3, 6.1, and 6.5% mass loss after 1, 2, and 4 weeks of incubation, respectively. Finally, the highest mass loss, with a small difference compared to the other two groups of samples, was observed in the results of the AkP30N group, where a mass loss of 1.7, 6.5, and 10.0% occurred after 1, 2, and 4 weeks of incubation, respectively. All groups presented low solubility values (<10%) after 28 days of incubation. After 28 days of incubation, the Ak scaffolds showed a complete collapse of the original 3D porous structure. However, the PLGA-coated akermanite scaffolds (AkP) and the akermanite scaffolds coated with PLGA containing nanoparticles at the highest concentration (AkP30N), despite the highest percentage of mass loss, preserved their 3D porous structure.

#### 3.3.6. Apatite-Forming Ability in SBF

**Figure 19:**
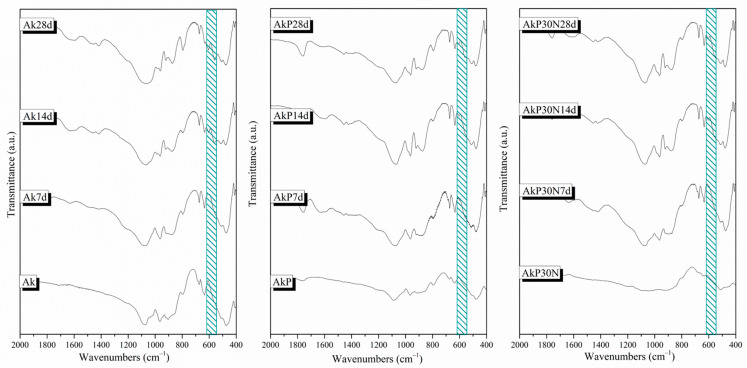
FTIR spectra of synthesized scaffolds after 1, 2, and 4 weeks of immersion in SBF. The shadow area between 560 and 640 cm^-1^ highlights the double peak attributed to the formation of an apatite layer on the surface of all samples.

After 7 days of immersion in SBF, a sharpening of the peaks attributed to the bioceramic structure (see Figure 19) was observed for the AkP and AkP30N scaffolds. This fact is due to the degradation of the polymeric coating on the surface of the samples (degradation rate of 1.3 to 1.7% wt). The presence of a shoulder at around 560 to 640 cm^−1^ was observed, which indicates the formation of an amorphous calcium phosphate phase on the surface of all scaffolds (Figure 19).

After 2 weeks of immersion, a sharpening of the broad peak at around 900–1200 cm^−1^ was observed, which is attributed to the bending vibration of the (PO_4_)^−3^ phosphate group. The presence of a double peak between 560 and 640 cm^−1^ indicates the formation of an apatite layer on the surface of the scaffolds (Figure 19). Finally, the peak around 1410–1510 cm^−1^ is also observed, which was not observed in any graph before immersion in SBF and which corresponds to the stretching vibration of the oxygen–carbon bond. The presence of this peak, combined with the small reduction in the silicate peak at 470 cm^−1^ and the formation of the double peak between 560 and 640 cm^−1^, indicates the development of a hydroxyapatite carbonate layer after 2 weeks of immersion (Figure 19) [27]. After 4 weeks of immersion in SBF, and while up to 10% degradation of the polymeric matrix occurs, a further sharpening of the double peak between 560 and 640 cm^−1^ is observed, indicating the enhanced growth of the hydroxyapatite layer on the surface of the samples.

**Figure 20:**
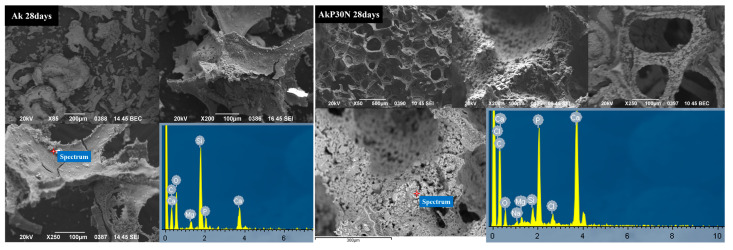
Micrographs of the akermanite (Ak) and composite akermanite/PLGA/NPs scaffolds (AkP30N) after 28 days of immersion in SBF.

As shown in the SEM micrographs after 28 days of immersion, a change in morphology was observed, which can be attributed to the formation of apatite layer on the surface of the scaffolds (Figure 20). EDS analysis after 28 days of immersion revealed that the average Ca/P molar ratios were 1.75, 1.71, and 1.68 for the Ak, AkP, and AkP30N scaffold groups, respectively, which are close to that ratio of stoichiometric apatite (≈1.67).

More specifically, pore wall collapse or fracture was observed in the micrographs of akermanite scaffolds without the PLGA coating (Ak). This is probably due to prolonged contact with the medium, as shown through the degradation results after 28 days of incubation that revealed the destruction of the three-dimensional porous structure. In contrast, SEM micrographs of the AkP and AkP30N scaffolds confirmed the preservation of the 3D porous structure. In addition, EDS results revealed the enhancement of the bioactive behavior of AkP30N scaffolds over AkP scaffolds, confirming that the enrichment of the polymeric matrix with bioactive nanoparticles improves the bioactivity of the scaffolds.

#### 3.3.7. Drug Loading and Release Studies

The encapsulation of MOX was estimated to be about 25.13% for a concentration of 1 mg/mL and 27.24% for the concentration of 3 mg/mL. It is obvious that a small difference is shown between the drug-loaded samples with different initial concentrations of the drug solution, which might be because of the certain solubility of moxifloxacin hydrochloride in the methanol where the drug loading process took part [44].

**Figure 21:**
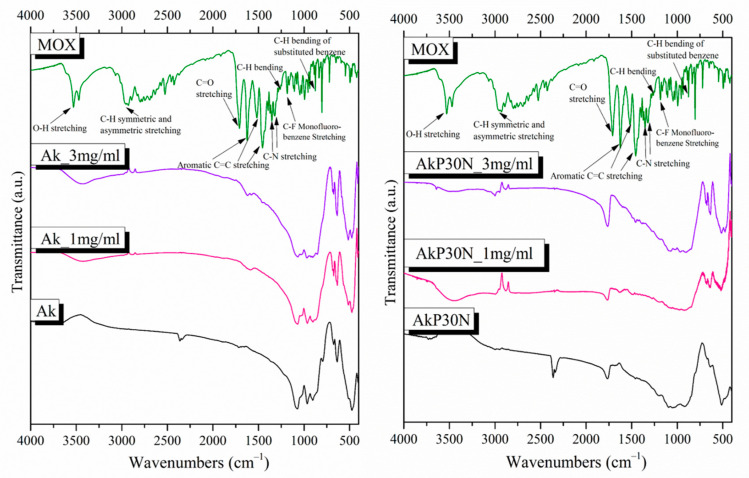
FTIR spectra of the scaffolds before and after drug encapsulation: akermanite ceramic scaffolds (Ak) and composite akermanite/PLGA/NPs scaffolds (AkP30N).

The presence of drugs in the akermanite-based scaffolds is confirmed through FTIR spectroscopy. The spectra of MOX-loaded and unloaded scaffolds, along with the spectrum of moxifloxacin as a reference, are presented in Figure 21. The spectrum of the MOX-loaded scaffolds presents the characteristic peaks corresponding to ΜOΧ, thus confirming the presence of the drug in them. The ((CH)–CH_2_), ν(NH_2_^+^), and δ_b_ (-CH_2_) vibrations are responsible for the peaks presented in the MOX spectrum at around 1415 to 1475 cm^−1^, 2520 cm^−1^, and 1356 cm^−1^, respectively. The peak at around 1620 cm^−1^ is presented due to the bending vibration of the N-H bond (presence of quinolones), the peaks at 802 and 991 cm^−1^ are attributed to C-H bond bending, and the peak at 1710 cm^−1^ corresponds to the vibration of the deformed δ_b_(COO-) bond [20].

**Figure 22:**
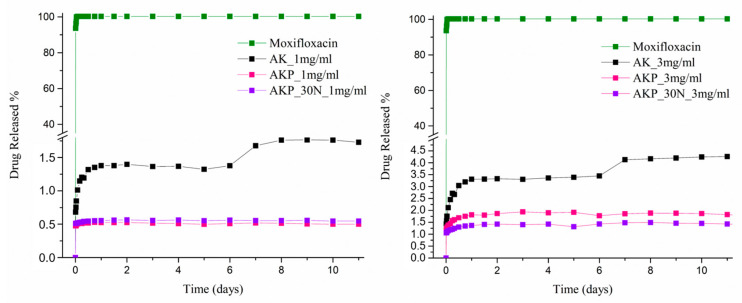
In vitro MOX release rate from the scaffolds at pH 7.4 for 1 and 3 mg/mL concentrations.

Figure 22 presents the results of the dissolution studies. Due to its hydrophilic nature (water solubility of 168 mg/mL), pure moxifloxacin was released in the PBS media and reached a plateau in less than 24 h. On the other side, in all cases of drug-loaded scaffolds, a more controlled release was obtained compared to the profile of the neat active substance, which is important for the prevention of infections and improvements in the bone regeneration process. More specifically, the drug-loaded neat Ak scaffolds displayed a biphasic release profile due to their initial burst release and subsequent sustained release after 6.5 days. The initial burst release, at first 12 h for both concentrations, is considered to be related to the drug absorbed and weakly linked on the surface of the scaffolds, while the extended release is related to the quantity of the drug mostly entrapped in their interior parts. The amount released from Ak scaffolds with 3 mg/mL moxifloxacin was higher than the Ak scaffold loaded with a moxifloxacin solution of 1 mg/mL, and this is related to the slightly larger drug loading percentage of the sample. To conclude, the incorporation of the drug into Ak improves its sustained administration. Concerning the Ak with the PLGA coating, it can be easily noticed that the use of a PLGA coating limits the initial burst release, as an even more controlled release appears compared to neat Ak-loaded scaffolds. In addition, the quantity of the released drug is smaller, as can be expected due to the polymer coating and its lower degradation in PBS media. Finally, the loaded Ak scaffolds coated with the NP polymeric coating presented similar release behavior at the 1 mg/mL loading concentration, while in the case of the 3 mg/mL concentration, a slower release rate was observed compared to the neat polymeric coating, probably due to interactions between the drug and the nanoparticles.

#### 3.3.8. Hemolysis Assay

**Figure 23:**
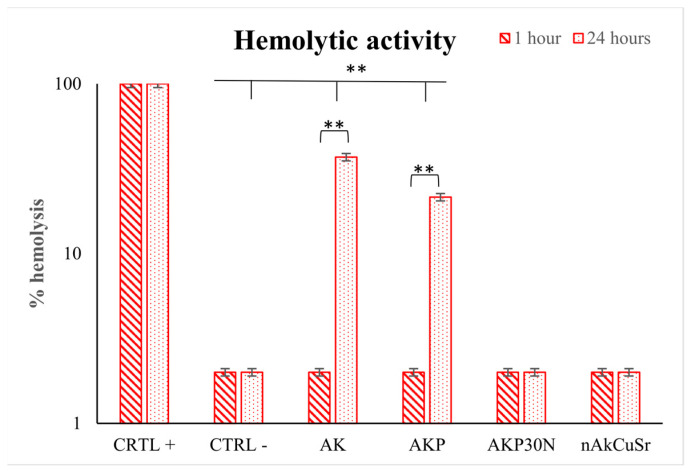
Hemocompatibility of scaffolds at body temperature after 60 min and 24 h of incubation. ** indicates statistically significant differences (*p* < 0.001) among negative controls and cells incubated with scaffolds, as well as between the incubation time points (1 and 24 h).

None of the tested scaffolds showed hemolytic behavior after 60 minutes of incubation with human erythrocytes (Figure 23). However, Ak and AkP scaffolds triggered hemolysis 24 h later compared to 60 min incubation (*p* < 0.001) (hemolysis rate of 37 and 21.5%, respectively) (Figure 23). The addition of NPs to the polymer coating did not result in hemolytic interactions and showed a fully hemocompatible profile (hemolysis rate of 2%).

## 4. Discussion

This work investigated the fabrication of composite 3D bioceramic/polymer scaffolds based on an akermanite bioceramic structure. The studied materials were fabricated with the goal of developing scaffolds for bone tissue engineering, a significant biomedical application that presents numerous material challenges. Bioceramic scaffolds such as akermanite have limited mechanical integrity; however, by combining synthetic degradable polymers, their fragility can be reduced. Synthetic degradable polymer PLGA with a well-defined structure and no immunological reactions was used to reduce fragility. Seven silica-based NPs were synthesized to be used as nanofillers in the PLGA matrix to mimic natural bone and enhance the biocompatibility and mechanical performance of akermanite scaffolds [45]. The nAkCuSr NPs were selected as fillers. As was mentioned in the results section, the seven nanobiomaterials did not present remarkable differences, remaining negatively charged and nanosized with high amorphous percentages and good hemocompatibility. However, the Sr and Cu co-doped NPs were selected for further study as they contain a unique composition that could enhance antibacterial capacity and angiogenesis, thus making them excellent candidates for tissue regeneration applications as functional fillers in composite polymeric scaffolds or coatings on the surface of implants.

Different bioactive powders were created using the sol–gel technique in the composition of akermanite to investigate the optimal conditions for the synthesis of the 3D bioceramic porous scaffolds. Akermanite is a crystalline phase of the ternary system SiO_2_CaOMgO, and, according to its phase diagram, presents a melting point at the temperature of 1454 °C, justifying the absence of a melting point in the studied temperature range [46]. The XRD results, in combination with the TG/DSC measurements, revealed that the optimal temperature to synthesize akermanite-based scaffolds is higher than 1300 °C, verifying that increases in temperature increase the percentage of akermanite crystallinity. Chengtie Wu et al. studied the formation of akermanite after sintering at 1100, 1200, and 1300 °C [30]. Study of the formation of crystalline phases using X-ray diffraction revealed the co-existence of merwinite and diopside, as well as the formation of the akermanite crystalline phase. However, the transformation of merwinite and diopside to akermanite was observed when the sintering temperature was increased. Furthermore, the same authors suggest 1300 °C as the temperature above which the merwinite and diopside crystalline phases can completely transform to akermanite. The transformation of merwinite and diopside into akermanite nanocrystals by increasing the temperature was also confirmed in a similar study by Choudhary et al. Those findings are in line with our findings [47]. In previous studies, it was also reported that it is possible for merwinite and diopside crystalline phases to form akermanite crystalline phase at a temperature above 800 °C, which is required for the formation to start according to the following equation [40]:

MgCaSi_2_O_6_ + Ca_3_Mg(SiO_4_)_2_ → 2Ca_2_Mg(Si_2_O_7_), ΔG_800°C_ = −18.06 kJ mol^−1^K^−1^ (4)

Gibbs free energy (ΔG) could help to predict the thermodynamic behaviors of chemical changes. The Gibbs free energy in the akermanite system at 800°C was determined by Myat Myat-Htun et al. (ΔG_800°C_ = −18.06 kJ mol^−1^K^−1^), which was estimated by fitting the Gibbs equation [40,48]. If the ΔG value is negative, the reaction is spontaneous (the reaction product is favored). If ΔG is positive, the reaction is nonspontaneous (reactant favored), and if ΔG is zero, the system is at equilibrium. The reaction presented a negative ΔG value, indicating the spontaneous formation of akermanite from a thermodynamic point of view. In full agreement with previous studies, the presence of a small percentage of merwinite and diopside, which are calcium magnesium silicate crystalline phases, is an expected finding. X-ray diffraction patterns also confirmed the transformation of these crystalline phases into akermanite with the increase in temperature [40,48]. Lower sintering temperatures can be used to improve the bioactivity of HAp bone grafts or scaffolds [49]. Keeping this in mind, we selected 1300 °C as a sintering temperature, the XRD results of which presented a high percentage of akermanite.

The ideal scaffold for bone engineering applications should have a porous structure with pores in the 200–500 μm range, which is essential for bone formation, neovascularization, and nutrient delivery [50]. In full agreement with the literature, the pore size of akermanite scaffolds was around 307 μm, while their mechanical properties were limited. As was observed in a previous study, PLGA scaffolds enhanced with hydroxyapatite (HAp) nanoparticles at 10–20% wt., possess higher compressive strength than neat PLGA scaffolds [51]. Previous studies have also shown that nanoparticles own high surface energy. Thus, the connection between the particles and the linear chains of the polymer is strengthened when nanofillers are added to the polymeric matrix [52]. In addition, the nanoparticles are wrapped by the linear chains of the polymer, which could lead to higher strength and cross-link junctions. Therefore, compressive strength increases with an increasing percentage of nanofillers in the matrix of PLGA [52]. This is also confirmed by our findings, as mechanical strength increased by up to almost 190% after application of the PLGA/NPs coating. Although compressive strength was increased, it is limited compared to the compressive strength of human cancellous bone, which is between 1.5 and 45 MPa [53]. This was expected as foam replica fabricated scaffolds present low compressive strength values. Similar findings were also observed by Wu et al. They fabricated akermanite scaffolds using the foam replica technique that presented compressive strength between 1.13 and 0.53 MPa [31]. The limited mechanical properties of foam replica scaffolds has driven attention to the development of ceramic scaffolds, which can be fabricated through the use of innovative alternative methods such as 3D printing [54]; 3D printing is one of the most promising methods for the development of biomimetic scaffolds for bone tissue engineering, but their mechanical strength is still limited. Advanced additive manufacturing techniques can be used to enhance the mechanical performance of ceramic scaffolds, such as using glass ceramics as a secondary filler to “heal” cracks and increase mechanical strength [55,56].

The successful regeneration of tissue depends in large part on the degradation of biomaterial scaffolds. Many biomedical applications, such as replacing hard tissues, require a scaffold to have the proper mechanical properties, which are typically on the same order of magnitude as those of the tissue it replaces. Additionally, the scaffold must degrade while retaining a particular minimal mechanical strength to support the formation of the new tissue. For example, in order to support bone regeneration, the material must be able to maintain its strength during the healing process, which, from a clinical standpoint, is typically at least 12 weeks [57,58]. In our study, the addition of nanofillers into the polymer coating (AkP30N scaffolds) increased the degradation rate, which was 10% after 4 weeks of study. In full agreement with these findings, previous studies revealed that the incorporation of bioactive nanofillers into a polymer matrix can prevent the collapse of the 3D porous structure of the composite scaffolds and increase the degradation rate. This fact is due to the percentage of added particles and the enhanced bioactivity due to their excellent bioactive behavior [45,59].

Τhe systemic toxicity of akermanite bioceramics was already investigated following well-accepted ISO standard methods in healthy adult rats. The injection of akermanite extracts in Wistar rats did not cause pathological changes to important organs, thus indicating the biosafety of akermanite bioceramics in clinical applications [60].

In a previous in vivo study, animals implanted with akermanite and composite akermanite/PCL scaffolds experienced severe hypothermia and weakness 48 h after implantation [61,62]. Hemolysis was then observed, and chemical analysis of the hemolytic blood revealed severe hypophosphatemia (<0.3 mg/dL) and phosphorus deficiency. Akermanite is a biocompatible ceramic that has been shown to improve stem cell adhesion, proliferation, and the maintenance of the osteogenic phenotype. However, in vivo implantation of two types of scaffolds resulted in acute toxicity and the disturbance of phosphorus homeostasis. This is due to the binding of phosphorus from the blood serum, thus creating hypophosphatemia and hemolysis [60]. To address the dose-dependent toxicity of akermanite, however, more research is required [61,62]. In our study, the addition of nanofillers to the polymeric coating of the composite scaffolds (AkP30N) did not induce hemolytic interactions and instead improved the hemolytic behavior of the scaffolds by creating a hemocompatible profile. PLGA is a highly hemocompatible biodegradable polymer [63]. Nevertheless, nanoparticles of the SiO_2_CaOMgO system doped with strontium and copper showed higher hemocompatibility, suggesting that this combination of ions is the most ideal for their use as nanofillers. This is confirmed by the increased hemocompatibility of the PLGA-coated akermanite scaffolds in which nanofillers from the nAkCuSr group were used within the polymeric coating. Our results are in accordance with other similar studies. Specifically, Nam et al. also used copper and strontium in TiO_2_ nanotubes to improve the hemocompatibility and the cytocompatibility of TiO_2_ implants for cardiovascular devices [64]. Additionally, strontium ion doping of mesoporous glass appeared to improve the hemocompatible profile of such materials for multifunctional biomedical applications [65]. In combination with the above, even if the high bioactivity of akermanite scaffolds can cause hypophosphatemia due to the rapid uptake of phosphorus from blood serum in the formation of an apatite layer, ultimately leading to hemolysis, it seems that the polymeric coating and the doping of NPs with copper and strontium improves hemocompatibility and prolongs the release of ions from the bioceramic structure (akermanite scaffold) upon interaction with the surrounding tissues and blood serum, thus reducing the severity of hemolytic effects.

Jadidi et al. investigated the encapsulation capacity and release rate of vancomycin from bredigite (Ca_7_Mg(SiO_4_)_4_) scaffolds before and after PLGA coating [43]. In their study, they found that the bredigite scaffolds without the PLGA coating showed the highest drug release rate (about 94.7 ± 2.5% of the encapsulated vancomycin was released within 9 h). However, they observed that the drug release rate after coating the scaffolds with PLGA was reduced beneficially in the first 6 h of release. It is important to mention that release of the drug in the first 6 h of implantation is necessary to prevent bacterial attachment and thus inhibit infections [66,67]. The authors observed that, after the initial burst of drug release, the rate of vancomycin release decreased, which was followed by a sustained release for the polymer-coated scaffolds. The akermanite scaffolds without the PLGA coating showed the highest drug release rate due to low degradation of the PLGA in composite scaffolds. Tailoring polymer blend/NPs percentage can help achieve more effective drug release. Staphylococcus aureus infections in the bone are a major issue in the biomedical field, and a new class of antibiotics must be investigated due to growing bacterial resistance [68]. Moxifloxacin is one of the most effective drugs against gram-positive bacteria, making it suitable for the treatment of bone infections [68,69]. It is administered orally in the form of 400 mg tablets and is recommended for patients before and after hip replacement surgery, as blood flow across the operated leg is constrained during knee replacement surgery [70]. Additionally, studies have been conducted to administer moxifloxacin or vancomycin to rat models for osteomyelitis. The experiment included treatment with 10 mg/kg BW moxifloxacin or vancomycin 10 mg/kg seven days after the experimental contamination. The authors concluded that, even if moxifloxacin was more sufficient against bacteria compared to vancomycin, the results of monotherapy were not as adequate as required [68].

An initial burst release is necessary to reduce the risk of infection and should be followed by a high enough drug concentration for an antibacterial effect in the recovery stage. Controlled and gradual release behavior was achieved in all cases for the scaffolds studied in this work [71]. In the present work, the loading capacity of ceramic structures at two different concentrations for local drug delivery was examined. The higher concentration (3 mg/mL) resulted in a higher loading percentage (~27.24%). This fact indicates the limited encapsulation capacity of the drug, which in absolute terms reveals that an increase in concentration leads to an increase in the encapsulation of MOX. For the Ak ceramic scaffolds, the quantity of drug released at the concentration of 1 mg/mL was 1.37% in the first 24 h and 3.31% at the concentration of 3 mg/mL, which can be translated into 0.092 mg and 0.544 mg of drug, respectively. Moreover, for the composite scaffolds (AkP30N), the quantity of drug released at the concentration of 1 mg/mL was 0.55% in the first 24 h and 1.37% at the concentration of 3 mg/mL, which can be translated into 0.024 mg and 0.21 mg of drug, respectively. However, according to the literature [72], the daily dose of antibiotics against osteomyelitis for a set of bacteria such as *S. aureus* (which grows during the early stages of implantation) is 0.06–128 µg/mL [72]. A minimum inhibitory concentration (MIC_90_) of 0.125 μg/mL has been reported for moxifloxacin against *S. aureus* [73]. This fact makes the composite scaffolds promising for use in bone tissue regeneration.

Additional research should be conducted in order to provide insights into the effects of the synthesized scaffolds on human mesenchymal stem cell (hMSC) attachment, proliferation, and multilineage differentiation, as well as the effects of coatings on the scaffolds.

## 5. Conclusions

Under the limitations of the present study, the following conclusions can be drawn:

- The different synthesized NPs presented sizes below 100 nm, negative ζ-potential, high ion incorporation, fast apatite formation, and good hemocompatibility without remarkable differences.
- The optimum temperature to synthesize akermanite-based scaffolds is ≥1300 °C, while increases in temperature increase akermanite’s crystallinity.
- The synthesized 3D porous akermanite scaffolds presented appropriate pore sizes for bone tissue engineering applications, while their mechanical properties were limited.
- The addition of nAkCuSr nanofillers improved compressive strength, hemocompatibility, and in vitro degradation while preserving the 3D porous structure and providing a more prolonged moxifloxacin release profile of.
- Akermanite scaffolds presented the highest MOX loading and release rates at the highest concentration, while MOX release from all scaffolds is efficient against osteomyelitis for a set of bacteria that grows during the early stages of implantation. This fact makes the composite scaffolds promising for use in bone tissue regeneration. Tailoring the amounts of bioceramic nanofillers and the degradation of PLGA coatings on akermanite scaffolds can be a promising strategy to develop composite multifunctional scaffolds for tissue engineering applications. Further in vitro and in vivo studies are needed to test the safety of the proposed scaffolds and their efficacy in bone regeneration.

## Figures and Tables

**Table 1:** Compositions of NPs in %mol.

Sample Code	SiO_2_	CaO	MgO	CuO	SrO
nSi	100				
nSiCa50	50	50			
nSiCa60	60	40			
nAk	40	40	20		
nAkCu	40	40	15	5	
nAkSr	40	40	15		5
nAkCuSr	40	40	15	2.5	2.5

**Table 2:** Identification and quantification (in %) of crystalline phases of nanobioceramics.

	nSi	nSiCa50	nSiCa60	nAk	nAkCu	nAkSr	nAkCuSr
Amorphous	100	51	75	49	38	43	36
Larnite		36	10		41	11	26
Ca_2_SiO_4_		13	6	25			
CaSiO_3_			9				
Magnesite				11		14	11
Monticellite				15			
Akermanite					4		
Magnesia					3		
Tenorite					2		2
Merwinite					12	12	
Ca_7_Mg(SiO_4_)_4_						13	
Diopside						4	
SrSiO_3_						3	
Sr_2_SiO_4_							2
Ca_5_MgSi_3_O_12_							23

**Table 3:** Results of ζ-potential measurements.

Sample Code	Size (nm)	ζ-Potential	PDI
nSi	281	−17.500	0.116
nSiCa50	396	−3.510	1.000
nSiCa60	342	−10.200	0.542
nAk	354	−13.600	0.489
nAkCu	220	−13.700	0.444
nAkSr	295	−17.400	0.438
nAkCuSr	259	−19.300	0.275

**Table 4:** Chemical composition of the synthesized NPs as detected by XRF in mol%.

Sample Code	SiO_2_		CaO		MgO		CuO		SrO	
	N ^1^	XRF	N ^1^	XRF	N ^1^	XRF	N ^1^	XRF	N ^1^	XRF
nSi	100	100								
nSiCa50	50	50.68	50	49.32						
nSiCa60	60	61.93	40	38.10						
nAk	40	39.40	40	42.86	20	17.74				
nAkCu	40	38.16	40	42.78	15	13.39	5	5.68		
nAkSr	40	40.14	40	42.81	15	12.79			5	4.27
nAkCuSr	40	39.10	40	42.59	15	13.33	2.5	2.89	2.5	2.10

^1^ N = nominal composition.

**Table 5:** Crystallization temperatures and mass loss (in %) at the main crystallization temperature.

Sample Code	Peak Τemperature of the Main Crystallization (°C)	(%) Mass Loss by the End of the Crystallization Process
BC60	910	73.5
BC700	871	1.9

**Table 6:** Quantitative analysis of the XRD patterns in %.

	BC700	BC960	BC1300	BC1400
Amorphous	14	8		
Ca_2_SiO_4_	66	54		
Akermanite			90	96
Merwinite	17	35	7	4
Diopside			3	
Periclase	3	3		

**Table 7:** Quantification analysis of akermanite scaffolds in %.

Crystalline Phase	Akermanite Scaffold (Ak)
Akermanite	93
Merwinite	4
Diopside	3

**Table 8:** Degradation rate (in %) of the synthesized scaffolds after 1, 2, and 4 weeks of incubation.

Mass Loss (%)			
	Ak	AkP	AkP30N
1 week	1.1	1.3	1.7
2 weeks	3.4	6.1	6.5
4 weeks	5.4	6.5	10.0

## Data Availability

All data are presented in the article.

## Supplementary Materials

The following supporting information can be downloaded at: https://www.mdpi.com/article/10.3390/pharmaceutics15030819/s1, Figure S1: Photograph of the akermanite composite scaffolds; Figure S2: Photograph of the scaffolds during the hemolytic activity of the scaffolds after 24 h of incubation; Figure S3: Photograph during the study of mechanical strength in ceramic and composite scaffolds.
