# Supplementary material for: Composite PLGA–Nanobioceramic Coating on Moxifloxacin-Loaded Akermanite 3D Porous Scaffolds for Bone Tissue Regeneration
Source: Pharmaceutics. 2023 Mar 2;15(3):819. doi: 10.3390/pharmaceutics15030819 (PMC10053907; doi:10.3390/pharmaceutics15030819)
Supplement: Supplementary file 1 [file pharmaceutics-15-00819-s001.zip › pharmaceutics-2213910-supplementary.pdf]

Supplementary Materials

S.1 Characterization of Ceramic Nanoparticles

S.1.1 X-Ray Diffraction (XRD)

A Ni-filtered CuKa radiation source and a Rigaku Ultima diffractometer (Rigaku Corporation, Tokyo, Japan) were used to conduct the XRD measurements. The operating parameters were: 2° scanning from 5° to 75°, step of 0.05°, and contact time of 1 s.

S.1.2 Fourier-transform infrared spectroscopy

FTIR spectra were collected in transmittance mode in mid-infrared region (4.000-400 cm^{-1}), by applying 2 cm^{-1} resolution and 32 scans using the Perkin Elmer Spectrum 1000 Spectrometer (Perkin Elmer Inc. , Waltham, MA, USA) To prepare the KBr pellets, a sample-to-KBr ratio of 1:100 was used and 7 tons pressure.

S.1.3 Particle Size Distribution and Z-potential Measurements by Laser Dynamic Light Scattering (DLS)

Particle size and Z-potential were measured by the dynamic light scattering (DLS) analyzer Zetasizer Nano Instrument (Malvern Instruments, NanoZS, ZEN3600, UK) using angle measurements of precisely 90° at 25 °C, using a 532nm laser. The samples were dispersed in an aqueous solution of KNO_3 (10 mM) and then filtered through 450nm pore filters after being sonicated at 25 °C. Each sample underwent an experiment in triplicate, and the ζ-potential value represents the mean average.

S.1.4 Scanning Electron Microscopy and Energy Dispersive Spectroscopic analysis (SEM-EDS)

The JEOL JSM-7610F Plus field-emission scanning electron microscope and an Oxford AZTEC ENERGY ADVANCED X-act energy dispersive X-ray spectroscopy (EDS) system (JEOL Ltd., Tokyo, Japan) were used to examine the morphological makeup of the samples. A 200Å thick carbon coating was applied to the samples to improve conductivity.

S.1.5 Transmission electron microscopy (TEM)

TEM images were obtained with FEI Tecnai G2 20 microscope at the accelerating voltage of 200 kV. The process for preparing TEM samples involved dissolving the powder in ethanol, dropping it on a grid covered in carbon, and drying it for two hours.

S.1.6 X-ray Fluorescence Spectroscopy (XRF)

A Bruker S4-Pioneer XRF wavelength dispersive spectrometer with an Rh tube and five analyzing crystals—LIF200, LIF220, LIF420, XS-55, and PET—was used to conduct a bulk analysis of the specimens. A scintillation detector, a gas-flow proportional counter, or both of them were used. Samples were examined under tube operating conditions of 60 kV and 45 mA. Lithium tetraborate (LiT or $Li_2B_4O_7$) was used as a flux during the fusion process to prepare the specimens as glass beads. The specimen-to-flux ratio was 1/8. A Vulcan fusion device (Fluxana, Bedburg-Hau, Germany) used a platinum crucible to fuse the mixture.

S.2 Characterization of Scaffolds

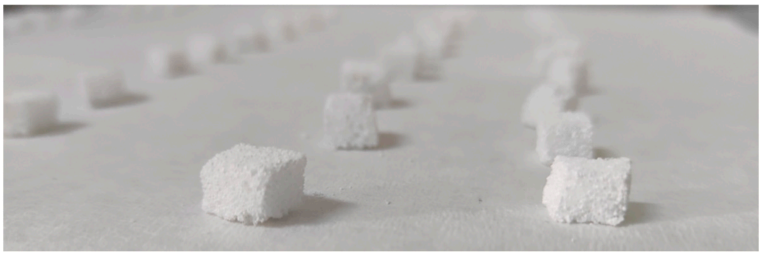

Figure S1: Photograph of the Akermanite composite scaffolds.

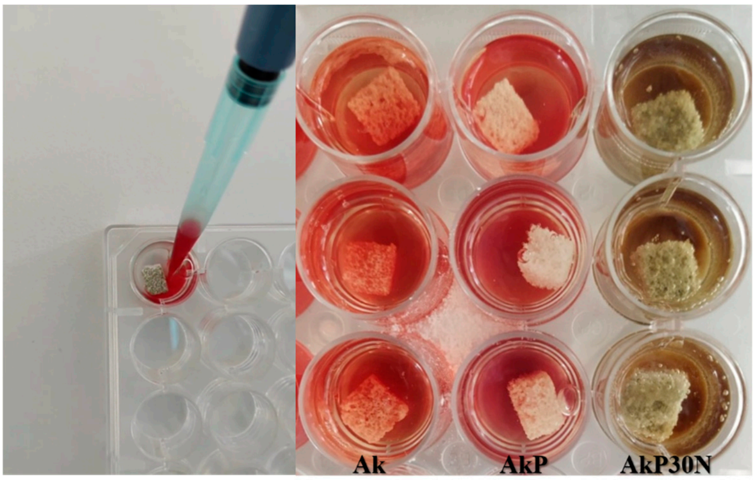

Figure S2: Photograph of the scaffolds during the hemolytic activity of the scaffolds after 24 hours of incubation.

S.2.1 FTIR Analysis of Scaffolds

In order to confirm the presence of akermanite crystalline phase and poly(lactic-glycolic) acid (PLGA) due to the polymer coating on the surface of the scaffolds, FTIR spectroscopy was used. A PerkinElmer Spectrometer (Spectrum 1000, Perkin Elmer Inc., Waltham, MA, USA) in transmittance mode in MIR (4.000-400 cm^{-1}), with a resolution of 2 cm^{-1}, and with 32 scans was used to conduct the FTIR analysis of the prepared scaffolds. Representative scaffolds from each sample were ground into a powder and used to create pressure-formed pellets with a sample-to-KBr mass ratio of 1:100.

S.2.2 XRD Analysis of Scaffolds

The XRD patterns of the synthesized scaffolds were conducted using a Rigaku Ultima diffractometer (Rigaku Corporation, Tokyo, Japan) with a Ni-filtered CuKa radiation source (= 0.1542). The operating parameters were the following: step of 0.05°, contact time of 1 s, and 2° scanning range from 5° to 75°.

S.2.3 SEM/EDS Measurements

Using the JEOL JSM-6390LV spectrometer, the morphology and microstructure of the scaffolds were examined using scanning electron microscopy (SEM) and an energy dispersive spectroscopic (EDS) analysis. The determination of mean pore size for each group of scaffolds was performed using ImageJ software.

S.2.4 Mechanical Properties

A universal testing machine (Instron 3344, Instron Int. Ltd., Norwood, MA, USA) is used to study the mechanical strength of the scaffolds in compression at a crosshead speed of 0.5 mm/min. More specifically, ten cubic scaffolds with dimensions of approximately $1 \times 1 \times 1$ cm^3 were tested. For each scaffold, the test lasted until 30% of compressive strain was reached. Stress-strain curves were obtained, while the mean values with the corresponding standard deviations were determined. The measurements were applied in accordance with ASTM D695 (ISO 604).

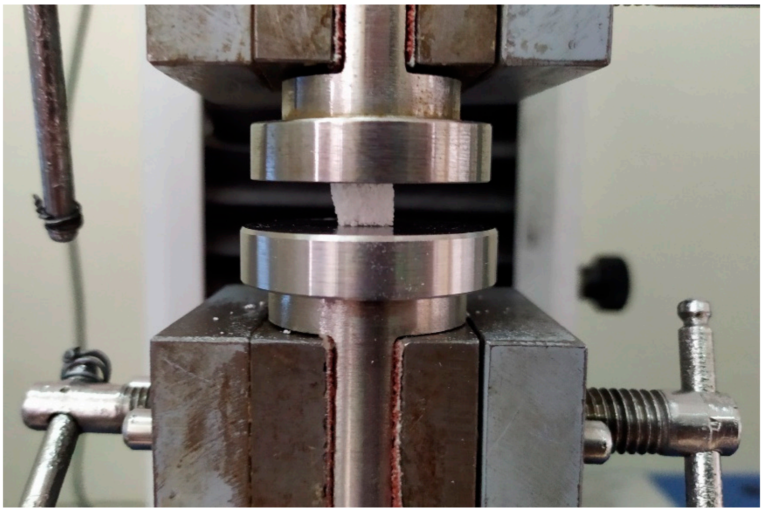

Figure S3. Photograph during the study of mechanical strength in ceramic and composite scaffolds.
